# Microbial Phosphorus Mobilization Strategies Across a Natural Nutrient Limitation Gradient and Evidence for Linkage With Iron Solubilization Traits

**DOI:** 10.3389/fmicb.2021.572212

**Published:** 2021-06-23

**Authors:** Shi Wang, Robert Walker, Marcus Schicklberger, Peter S. Nico, Patricia M. Fox, Ulas Karaoz, Romy Chakraborty, Eoin L. Brodie

**Affiliations:** ^1^Ecology Department, Climate and Ecosystem Sciences Division, Earth and Environmental Sciences Area, Lawrence Berkeley National Laboratory, Berkeley, CA, United States; ^2^School of BioSciences, University of Melbourne, Parkville, VIC, Australia; ^3^Energy Geosciences Division, Earth and Environmental Sciences Area, Lawrence Berkeley National Laboratory, Berkeley, CA, United States; ^4^Department of Environmental Science, Policy and Management, University of California, Berkeley, Berkeley, CA, United States

**Keywords:** phosphorus limitation, phosphorus mobilization, microbial traits, iron complexed organic phosphorus, siderophores

## Abstract

Microorganisms have evolved several mechanisms to mobilize and mineralize occluded and insoluble phosphorus (P), thereby promoting plant growth in terrestrial ecosystems. However, the linkages between microbial P-solubilization traits and the preponderance of insoluble P in natural ecosystems are not well known. We tested the P solubilization traits of hundreds of culturable bacteria representative of the rhizosphere from a natural gradient where P concentration and bioavailability decline as soil becomes progressively more weathered. Aluminum, iron phosphate and organic P (phytate) were expected to dominate in more weathered soils. A defined cultivation medium with these chemical forms of P was used for isolation. A combination of soil chemical, spectroscopic analyses and 16S rRNA gene sequencing were used to understand the *in situ* ability for solubilization of these predominant forms of P. Locations with more occluded and organic P harbored the greatest abundance of P-mobilizing microorganisms, especially *Burkholderiaceae* (*Caballeronia* and *Paraburkholderia* spp.). Nearly all bacteria utilized aluminum phosphate, however fewer could subsist on iron phosphate (FePO_4_) or phytate. Microorganisms isolated from phytic acid were also most effective at solubilizing FePO_4_, suggesting that phytate solubilization may be linked to the ability to solubilize Fe. Significantly, we observed Fe to be co-located with P in organic patches in soil. Siderophore addition in lab experiments reinstated phytase mediated P-solubilization from Fe-phytate complexes. Taken together, these results indicate that metal-organic-P complex formation may limit enzymatic P solubilization from phytate in soil. Additionally, the linked traits of phytase and siderophore production were mostly restricted to specific clades within the *Burkholderiaceae*. We propose that Fe complexation of organic P (e.g., phytate) represents a major constraint on P turnover and availability in acidic soils, as only a limited subset of bacteria appear to possess the traits required to access this persistent pool of soil P.

## Introduction

Phosphorus (P) is one of the most limiting plant nutrients, with yield on 30–40% of the world’s arable land limited by P availability (Runge-Metzger, 1995; Nesme et al., 2018). During soil formation and aging (pedogenesis), P weathers from a mineral form to a labile form (dissolved orthophosphate, PO_4_^3–^) that is available for plant uptake. However, much of the labile P becomes sorbed onto mineral surfaces, immobilized into soil organic matter, or incorporated into recalcitrant inorganic forms. These processes result in eventual depletion of labile P with an accumulation of the occluded and organic forms of P that are not readily accessible to plants (Izquierdo et al., 2013; George et al., 2018). The organic P fraction of which phytate (inositol phosphate) is frequently a major component (McKercher and Anderson, 1989; Turner et al., 2006), can account for 20–95% of total P (Dalai, 1977; Stewart and Tiessen, 1987). The fixation and subsequent immobilization of phytate in soil is often much stronger than that of the orthophosphate anions (McKercher and Anderson, 1989), however, the reasons for the accumulation of phytate in soil are not fully clear. Owing to its high negative charge, phytate is tightly adsorbed to various soil components (Lung and Lim, 2006) and the sorption of phytate to soil solid phases has been shown to induce the formation of Fe/Al-phytate precipitates at oxide surfaces (Celi and Barberis, 2005; Yan et al., 2014), or the formation of humic-Fe(Al)-phytate complexes at humic surfaces via Fe or Al bridges (He et al., 2006; Gerke, 2010; Chen et al., 2018).

To access P of various forms, plants are aided by the growth and activity of soil microorganisms, which possess metabolic mechanisms to mineralize and mobilize plant inaccessible P into orthophosphate by (1) enhanced dissolution of P containing minerals through soil acidification or release of metal chelating ligands e.g., organic acids, siderophores, etc. (Khan et al., 2014; Alori et al., 2017); (2) secretion of extracellular enzymes to mineralize organic P (Rodríguez and Fraga, 1999; Sharma et al., 2013; Alori et al., 2017), and (3) reductive dissolution of Fe(Al)-complexed organic P (He et al., 2006; Chen et al., 2018). Although, a number of organisms e.g., *Pseudomonas*, *Burkholderia*, and *Bacillus* spp. have been identified as phosphate-solubilizing bacteria (PSB), largely based on laboratory experiments (Tarafdar and Claassen, 1988; Rodríguez and Fraga, 1999; Olander and Vitousek, 2000; Chen et al., 2008; Oteino et al., 2015; Wang et al., 2017), their modes of action under *in situ* conditions has not been well documented (Vessey, 2003; Jilani et al., 2007; Timmusk et al., 2017) resulting in uncertainty as to their actual contributions to P mobilization. The complex interactions that occur between the microbial metabolic or enzymatic products, the chemistry of soil minerals, the aqueous phase and organic matter, in addition to the chemistry and physiology of plant roots makes it challenging to decipher *in situ* mechanisms (Jones and Oburger, 2011). For example, phytic acid can be strongly bound to soil minerals and may result in insoluble Fe(Al)-complexed phytic acid (He et al., 2006; Gerke, 2010). Enzymatic microbial hydrolysis of phytic acid requires the binding of free phytic acid to the substrate-binding pocket of phytase enzymes; hence, once tightly sorbed via Fe and Al bridges, or co-precipitated as a metal-organic P complex it is less susceptible to direct enzymatic hydrolysis (Veinot and Thomas, 1972; Dao, 2003; He et al., 2006). Therefore to access P in Al/Fe complexed phytic acid, microbes likely need to coordinate multiple metabolic strategies such as ligand and enzyme secretion to mobilize insoluble P.

To date, research has focused more on the biological availability of inorganic rather than organic P (Jones and Oburger, 2011; Gerke, 2015; Alori et al., 2017). However, both soil pH and soil mineralogy are key regulators of P solubility and the study of microbial P solubilization must consider the primary *in vitro* forms of P to which the indigenous microbiome may have adapted. Our study focused initially on the mobilization of P from both organic (phytate) and inorganic (aluminum and iron phosphate) forms that were expected to dominate at our study site, termed “The Ecological Staircase.”

The “Ecological Staircase” (ES) in Mendocino County, California also known as the Hans Jenny Pygmy Forest Reserve, is part of the University of California, Natural Reserve System. It represents a soil chronosequence exhibiting extreme nutrient limitation across a gradient comprised of five wave-cut marine terraces (T1–T5) formed through wave-action and elevated through tectonic activity (Jenny et al., 1969; Westman and Whittaker, 1975; Yu et al., 2003; Izquierdo et al., 2013). Soils on the older terraces (T3–T5) have been intensively weathered, becoming enriched in Fe and Al oxides resulting in highly leached spodosols and ultisols with lower fertility and higher acidity than the younger terraces dominated by inceptisols and ultisols (Northup et al., 1998; Yu et al., 2003). Consequently, soil development and therefore the distribution of P between the organic and inorganic pools, as well as the composition of P forms, have a major impact on P accessibility for both plants and microorganisms. Such a gradient over a relatively short distance (∼3 miles) represents an ideal system to study the adaptation of soil microbes in response to severe nutrient stress.

Previous work investigating P availability at the Ecological Staircase (Northup et al., 1995; Izquierdo et al., 2013; Uroz et al., 2014) set the basis for our study. With an accumulation of the occluded and organic forms of P, P becomes a primary limiting nutrient in older terraces. Uroz et al. (2014) addressed the relationship between nutrient availability and taxonomic and functional structure of the soil bacterial communities. They also determined the P solubilization capabilities of culturable soil bacteria, using tricalcium orthophosphate as the sole P source. However, due to the increasing acidity with weathering across the terraces, insoluble calcium forms of P are not expected to be as relevant here beyond terrace 1. For our work we focused on the mobilization of P from aluminum phosphate (AlPO_4_), iron phosphate (FePO_4_), and organic P (phytate) expected to dominate here (Izquierdo et al., 2013). Our goal was to determine the range of culturable bacteria, representative of the rhizosphere, that were capable of solubilizing these forms of P across the nutrient limitation gradient and develop an understanding of P mobilization mechanisms. We used a combination of soil chemical and spectroscopic analyses and 16S rRNA gene sequencing to understand the *in situ* chemical and microbial context of these isolates respectively. Here we show that the capacity to solubilize different insoluble P forms varies across microbial phylogenetic groups and by location across the gradient. We propose that Fe complexation of organic P, like phytate, may represent a major constraint on P turnover and availability in these soils, and that only a subset of organisms possess the traits required to access this important pool.

## Materials and Methods

### Overview

We sampled across three terraces (T1–T3) at the Ecological Staircase in Mendocino, CA, United States. We characterized soil chemistry by measuring soil pH, exchangeable cations, cation exchange capacity (CEC), extractable iron (amorphous and crystalline iron-oxides), and extractable P. In addition, micro X-ray fluorescence (μ-XRF) was applied to determine the physical associations of P with other elements. 16S rRNA gene iTag sequencing was used to determine microbial community composition, diversity and relative abundance as context for cultivated organisms and we used quantitative PCR to quantify bacterial/archaeal abundances across the gradient. A defined cultivation medium with AlPO_4_, FePO_4_, or phytate as sole P sources was used in order to isolate PSB. Full-length 16S rRNA gene sequencing was used to identify and phylogenetically place these PSB. We characterized phenotypic traits of these PSB, measuring P solubilization colorimetrically as well as testing for siderophore production as a key iron solubilization trait. Subsequent experiments were also conducted to determine the influence of metal complexation on enzymatic organic P solubilization. Detailed methods are provided below.

### Site Selection and Sample Collection

The study site (Ecological Staircase) in Jug Handle State Natural Reserve (39_ 220 310 N, 123_ 480 370 W) is located on the Mendocino coast, California, about 240 km north of San Francisco. We sampled the soil chronosequence across five wave-cut marine terraces (T1–T5) formed through wave-action and elevated through tectonic activity. This “staircase” where each steps corresponds to approximately 100,000 years of soil development and weathering is characterized by extreme nutrient limitation. Terraces T1 and T2 display tall bishop pine (*Pinus muricata*) forests with mature trees more than 20 m in height, whereas mature trees of Mendocino cypress (*Cupressus pygmaea*), no more than 5 m tall are found on the oldest, most acidic soils T3, T4, and T5 (Northup et al., 1995; Westman and Whittaker, 1975). Total soil P steadily decreases from T1 to T3, remaining nearly constant from T3 to T5 (Izquierdo et al., 2013). Labile P, representing inorganic P that is readily accessible to plants, decreases steadily from T1 to T5, while soil organic P declines consistently across the first four terraces, with only a slight increase on T5 (Izquierdo et al., 2013). The organic and occluded P forms, which are less accessible to plants, become proportionally more abundant as the soils age (Izquierdo et al., 2013). T3, T4, and T5 share similar vegetation communities and similar soil types and properties but differ in age. For this study, we focused on terraces T1, T2, and T3 (Supplementary Table 1).

Soil samples were collected from each of the three terraces in November 2014. An auger with plastic liners (5 cm diameter) and caps was used to collect four cores per terrace. Sub-sampling locations on the same terrace were approximately 10 m apart. Four soil cores collected on T1 (N 39°22.627′_W 123°48.884′) were excavated up to 18 cm in depth. Soil cores on T2 (N 39°22.643′_W 123°48.340′) were up 5 cm in depth. Cores from T3 were up to 25 cm in depth (N 39°22.383′_W 123°46.856′). Soil cores were kept on blue ice and transported back to the lab, after which field–moist soil samples from T1 (0–18 cm) and T2 (0–5 cm) with visibly uniform A horizons were separately homogenized. During field sampling, distinct zonation visible as color transitions was observed in the T3 soil cores from a white/ash colored layers on the surface to earth brown and yellow colors deeper into the profile. Thus, the T3 soil cores were sectioned by depth based on these color transitions and labeled accordingly as T3 (0–7 cm), T3 (7–13.5 cm), T3 (13.5–20 cm), and T3 (20 cm-end). After separate homogenization of each section from each terrace/core, 5 g soil for each sample were stored at −80°C for later molecular biology work and the remainder stored at 4°C for microbiological and chemical analyses.

### Soil Chemical Characterization

Two of the field replicates were subsampled for chemical analysis. The chemical analysis data presented is the average of two field replicates (not technical replicates). Soil samples were analyzed for pH, exchangeable cations, CEC, extractable iron (amorphous and crystalline iron-oxides), and extractable P. The pH measurements were performed by mixing 2.5 g field-moist soil with 2.5 mL deionized water and the pH was measured in the slurry after 15 min. Exchangeable cations and CEC were measured using the compulsive exchange method (Sumner and Miller, 2018). The CEC is determined by the loss of Mg in the final solution. Soil samples were extracted with a solution of 0.25 M NaOH and 0.05 M disodium EDTA in order to extract organic and moderately bound inorganic P according to Cade-Menun and Preston (1996). Approximately 3 g of field moist soil was extracted with 25 mL of NaOH-EDTA solution for 5 h. The samples were then centrifuged and the supernatant filtered through a 0.45 μm PVDF syringe filter. An aliquot of the filtered extract was diluted and analyzed for total P by inductively coupled plasma mass spectrometry (ICP-MS) (Perkin-Elmer Elan DRC II).

A subsample of soil was air-dried and ground to a fine powder in a ball mill using tungsten-carbide balls prior to extraction of iron. The amorphous iron oxide content was determined by extraction with ammonium oxalate according to (Loeppert and Inskeep, 1996). The total free iron oxide content was determined by the citrate-bicarbonate-dithionite (CBD) method, wherein 0.5 g of sediment was extracted with 0.3 M sodium citrate, 0.1 M sodium bicarbonate, and sodium dithionite (added in two 0.5 g portions) at 80°C (Loeppert and Inskeep, 1996). The extracts were then filtered through a 0.45 μm PVDF syringe filter and analyzed for Fe and Al.

All solution extracts were analyzed by ICP-MS after dilution in ultra-pure 0.15 M nitric acid and internal standard addition.

### Micro X-Ray Fluorescence (μ-XRF)

Soil samples from T1 to T3 (one field replicate for each) were prepared into thin sections and analyzed by μ-XRF in order to determine elemental associations. Subsamples of soil were air-dried, embedded into epoxy (EPO-TEK 301-2FL, Epoxy Technology, Inc.), and allowed to cure under a vacuum for 3 days. Once cured, the resin block was shipped for thin section preparation (Spectrum Petrographics, Inc., Vancouver, WA, United States). Standard thin sections (30 μm thick) were mounted onto quartz slides. Thin sections were analyzed by μ-XRF at beamline 10.3.2 at the Advanced Light Source at Lawrence Berkeley National Lab. Maps were collected at energies of 2152.3 and 7200 eV with dwell times of 200 and 50 ms per point for P and Fe, respectively. Pixel size ranged from 20 to 50 μm. The X-ray energy was calibrated to the white line of a sodium phosphate Na_3_PO_4_ at 2152.3 eV. Fluorescence maps were analyzed using LabView. We analyzed one field core per terrace for T1, T2, and T3 (in addition to multiple depth intervals for T3). For each surface sample we collected multiple maps (including visual microscope images, coarse scale maps and fine scale maps). We used the coarse maps (pixel size 50 μm, 100 ms dwell time) to identify regions where P was observed (hot spots), focusing on those P spots with fine scale maps (20 μm pixel size, 200 ms dwell time). We collected fewer maps from the deeper T3 samples (2–5) because very few P spots were observed. Those regions that were most completely analyzed are shown in the included figures. These were selected based on qualitative assessment and they were representative of what was seen on the coarser scale, larger maps.

### Soil Genomic DNA Extraction and 16S rRNA Gene Barcoded Sequencing and Analysis

Total DNA was extracted from each sample by using 0.5 g soil as input into the PowerSoil DNA isolation Kit (Mo Bio, Carlsbad, CA, United States) with minor modification as described before (Sorensen et al., 2020). Three DNA extraction controls were included. DNA amount was quantified by using Qubit dsDNA HS assay (Invitrogen, Carlsbad, CA, United States). The V3–V4 region of bacterial 16S rRNA gene was amplified using the primers 338F (5′-ACT CCT ACG GGA GGC AGC A-3′) and 806R (5′-GGA CTA CHV GGG TWT CTA AT-3′) (Mori et al., 2014). PCR amplification was conducted in 25 μl reactions containing a final concentration of 1× Takara Ex Taq buffer, 0.625 U/ul Ex Taq polymerase, 1 μg/μl BSA, 200 μM Takara dNTP mix, 15 ng/DNA template, and 1000 pM primers. The 16S rRNA gene fragments were amplified under the following conditions: an initial denaturation at 95°C for 10 min, 30 cycles of 95°C for 30 s, 52°C for 40 s, and 72°C for 90 s, and a final extension at 72°C for 10 min. We included three extraction controls in PCR amplification in addition to PCR negative controls. There was no amplification shown in these controls as assessed by both 2% agarose gel and Bioanalyzer. These controls did not move forward for sequencing analysis. The triplicate PCR products were then pooled and purified with the use of Sera-Mag carboxylate-modified magnetic particles (Thermo scientific, Fremont, CA, United States) and sequenced on one lane of the Illumina MiSeq paired end with 150 cycles. Quality filtering and demultiplexing were performed by Vincent J. Coates Genomics Sequencing Laboratory at UC Berkeley (Berkeley, CA, United States).

Pairs of forward and reverse reads (with bases > Q30) were aligned using usearch (v8.1.1861) (Edgar, 2013) *fastq_mergepairs* (16S: fastq_maxdiffs = 3). The resulting aligned reads were quality filtered with usearch *fastq_filter* (16S: trunclength = 250, truncqual = 2, maxee = 0.5). Separately for each amplicon, quality filtered and corrected sequences from all samples were concatenated, dereplicated with usearch *derep_fulllength*, sorted and rare sequences were removed with usearch *sortbysize* (minsize = 5). The resulting sequences were used for OTU clustering at 97% identity with usearch *cluster_otus* (otu_radius_pct = 3) and OTU representative sequences were saved. Chimeric sequences were filtered with usearch *uchime_ref* (Edgar, 2013) using the following reference databases (16S)^1^. OTU abundances across individual samples were calculated by mapping chimera-filtered OTUs against the quality-filtered reads using usearch *usearch_global* command (id = 0.97, strand = plus) and the resulting OTU table was saved. Taxonomy was assigned to OTUs using OTU representative sequences and a Naïve Bayes classifier as implemented in R dada2 package (Callahan et al., 2016) (*assignTaxonomy*, minBoot = 60) trained with the following reference sequences: 16S: SILVA release 132).

Raw de-multiplexed sequences have been archived in the NCBI Bioproject database and are publicly available under accession no. PRJNA643303.

### Quantitative PCR

Quantitative PCR (qPCR) assays were used to demine the total copy number of bacterial/archaeal 16S rRNA genes. These assays were performed on extracted soil DNA carried out in a Bio-Rad iCycler single-color real-time PCR detection system (Bio-Rad, Hercules, CA, United States). The 20 μL reaction volume contained 10 μL using universal SYBR Green supermix (Bio-Rad, Hercules, CA, United States), 1 μL of each primer (500 nM stocks) and 5 ng of DNA template. The primers, standards construction, and PCR conditions were described in Supplementary Materials. The qPCR assays were performed in triplicate for each sample in 96-well plates. After confirming the presence of target PCR product in each reaction using a melting curve analysis with the MyIQ software (Bio-Rad, Hercules, CA, United States), standard curves were used to calculate target copy numbers for each reaction. Controls without DNA template controls were included in every run.

### Isolation Media With Insoluble P Forms

NM8 cultivation media was amended with AlPO_4_, FePO_4_, or phytic acid sodium salt (phytic acid) as the sole P source and common plant exudate carbohydrates as carbon and energy sources. NM8 liquid medium (Illmer and Schinner, 1992) was modified as follows: NH_4_NO_3_, 373 mg; MgSO_4_.7H_2_O, 410 mg; KCl, 295 mg; NaCl, 200 mg; glucose, 2 g; sucrose, 2 g; and fructose, 2 g, DL minerals without NTA 10 ml, DL vitamin 10 ml and up to 1000 ml with milliQ water pH6 ± 0.2 (MES buffered). After autoclaving, different sole P sources were added at a final concentration of 0.5 mM. For agar media, 15 g Noble agar L^–1^ were added to the medium.

To remove any potential phosphate residue on glassware, all glassware used for this experiment were washed with 1% solution of hydrochloric acid and rinsed with DI water three times.

### Isolation of Potential Phosphate Solubilizing Bacteria and Taxonomic Characterization

All soil samples (four field replicates per terrace) were used for isolation. For each soil sample, 2 g of soil were dissolved in 10 ml of a cell dislodging buffer containing a final concentration of 0.05% Tween80, 0.2 M NaCl, 0.05 M Tris HCl and MilliQ H_2_O and shaken for 16 h. After shaking, the soil slurry was vortexed at maximum speed for 10 min and centrifuged at 1000 rpm. Suitable dilutions (50 μL) of each original soil suspension were inoculated on modified NM8 medium agar plates containing either 0.5 mM FePO_4_ or 0.5 mM AlPO_4_ or 0.5 mM phytic acid as the sole P source (Illmer and Schinner, 1992). Plates were incubated at 28°C for 5 days, following which, a total of 288 colonies (96 from each P source) were selected on the basis of different colony morphology (shape, texture, edge as well as color of the colonies) for further colony purification in the same medium. Once determined pure, axenic colonies were transferred from plates into corresponding liquid medium with the appropriate P sources.

DNA was extracted from the cultures using the MoBio UltraClean Microbial DNA isolation Kit (Mo Bio Inc., Carlsbad, CA, United States). PCR amplification was conducted using universal bacterial 16S rRNA gene primers 27F (5′-AGA GTT TGA TCM TGG CTC AG-3′) and 1492R (5′-GG TTA CCT TGT TAC GAC TT-3′) (Frank et al., 2008). PCR amplification was conducted in 25 μl reactions containing a final concentration of 1× Takara Ex Taq buffer, 0.625 U/ul Ex Taq polymerase (Takara Bio, Mountain View, CA, United States), 1 μg/ul BSA, 200 μM Takara dNTP mix, 15 ng/DNA template, and 0.2 μM primers. The PCR program used was the same as described above. Each sample was amplified in triplicate, combined and purified using the Sera-Mag carboxylate-modified magnetic particles (Thermo scientific, Fremont, CA, United States). Sequencing samples were prepared with 20 ng PCR product and 4 pmol primer and sequenced using Sanger sequencing at California University of Berkeley Sequencing Facility. Sequences were visualized using 4 Peaks and edited using Seaview (Gouy et al., 2010) and trimmed to remove bases below a phred score of 45 as necessary. The 16S rRNA sequences were aligned using Geneious software (Kearse et al., 2012) with corresponding reference sequences from NCBI, and phylogenetic analysis was carried out using maximum parsimony criteria in MEGA6 software (Kumar et al., 2008). Heuristic tree searches were performed using a tree bisection reconnection model and a branch-swapping algorithm with 100 random stepwise swaps. One hundred trees were calculated for each pseudo replicate. A rescaled consistency index, derived from trees, was used to generate an *a posteriori* weighted data set. The same heuristic search conditions were used to analyze the weighted data set. Branch support was obtained with 100 bootstrap replicates. *Desulfurobacterium thermolitotrophum* (NR_025270) was used as an out-group in each phylogenetic reconstruction. A phylogenetic tree was visualized using ITOL tree (Letunic and Bork, 2016).

Full length 16S rRNA sequences have been archived in the NCBI GenBank database and are publicly available under accession no. MT706793-MT707062.

### High Throughput Phosphate Solubilization Assays

The ability of all bacterial strains to solubilize FePO_4_, AlPO_4_, and phytic acid was determined by the malachite green colorimetric assay using a QuantiChrom phosphate assay kit (BioAssay Systems, Hayward, CA, United States). Isolates grew in 96 well plates with different P sources for 96 h. Cultures were centrifuged and supernatants were filtered through a 0.22 μm PVDF filter plate using MultiScreen_HTS_ vacuum manifold system (EMD Millipore, Merck KGaA, Darmstadt, Germany). 50 μL filtered supernatants were used for phosphate assays by following the QuantiChrom kit protocol. Medium blanks and calibration standards were analyzed at the same time. The absorbance at 620 nm was measured using a SPECTRAmax PLUS 384 spectrophotometer (Molecular Device, Sunnyvale, CA, United States).

### High Throughput Siderophore Production Assays

An overlay Chromeazurol S assay (O-CAS assay) was used to detect siderophores produced by bacterial isolates. The composition of the overlay CAS blue agar medium for this assay was prepared according to Pérez-Miranda et al. (2007). Twenty microliters of each isolate grown to mid-log phase were inoculated on 48-well culture plates containing 800 μL/well NM8 medium agar with NaH_2_PO_4_ as the sole phosphate source (pH 6 ± 0.2) and incubated at 28°C for 72 h (all isolates formed biomass). Post-incubation, 300 μL overlays of CAS were applied over each well, and incubated overnight in the dark. Siderophore production was indicated by a change in color observed in the overlaid agar from blue to purple or to reddish orange or to yellow. As reported, different colors represent different type of siderophores, purple indicates catechol type production, yellow indicates hydroxamate type production and reddish orange indicates a mix of different type of siderophores (Schwyn and Neilands, 1987; Pérez-Miranda et al., 2007). Siderophore production was quantified by the size of halo in millimeters.

### Assaying the Influence of a Microbial Siderophore on the Hydrolysis of Phytic Acid by Phytase Enzymes in the Presence and Absence of Iron

To evaluate the influence of iron complexation and co-precipitation on the ability of phytase (Sigma-Aldrich, CAS Number 9001-89-2) to hydrolyze phytic acid, an Fe-phytate complex (phytate:ferric complex) was created by adding 1 mM phytate and 2.5 mM ferric iron in the form of FeCl_3_. The precipitated complex was centrifuged at 10,000 × *g* for 5 min and the supernatant discarded to remove excess chloride ions and non-complexed Fe and phytic acid. The phytate:ferric pellet was resuspended in sodium acetate reaction buffer (27.22 g/L sodium acetate; pH 5.15) to assay PO_4_^3–^ release from phytate, with all reactions carried out in 5 ml reaction buffer. We were particularly interested in the ability of siderophores to solubilize complexed Fe and potentially improve phytase hydrolysis of phytate:ferric complexes. The experimental treatment for this (phytate:ferric complex + phytase + siderophore) contained 5 μmol (1 mM) of phytate, 12.5 μmol (2.5 mM) of iron FeCl_3_ and 5 μmol (1 mM) of ferrichrome with 40 mg/ml phytase. The relevant experimental controls were phytate + phytase; and phytate:ferric complex + phytase. Each reaction was incubated at 55°C, 500 μL samples were collected at 7.5 min intervals and 500 μL of 0.5 M NaOH was used to terminate the reaction by enzyme denaturation, this also solubilized the phytate:ferric complex. Phytate and PO_4_^3–^ concentrations were then determined by Dopmex-2100 ion chromatography with Dionex IonPac AS11-HC IC Column (Thermo Fisher Scientific, Waltham, MA, United States).

### Statistical Analysis

All statistical analyses were completed using the R v3.3.1 statistical programming environment (R Development Core Team, 2010). Sample metadata, OTU tables, taxonomic assignments, and representative OTU sequences were imported as a phyloseq (McMurdie and Holmes, 2013) object into R. Using the phyloseq object created through the above pipeline, OTUs with relative abundance less than 0.1% across all samples per location were filtered pre-comparison. Wilcoxon-tests for OTUs after filtering were performed across locations.

The differences between gene copy numbers, phylogenetic diversity (Faith’s PD), relative abundances of taxonomic families across terraces and P concentration solubilized from different P sources by bacteria isolates obtained across terraces were tested for significance with Kruskal-Wallis test (R base implementation), followed by *post hoc* Mann-Whitney-Wilcoxon-tests (non-parametric) to interpret pairwise differences.

Microbial communities characterized using 97% homology OTUs were ordinated using non-metric dimensional scaling (NMDS) based on weighted phylogenetic distances (weighted UniFrac distance) as implemented in the phyloseq package (McMurdie and Holmes, 2013). Differences in community structure across groups were evaluated with permutational multivariate ANOVA [implemented in PERMANOVA in vegan package (Oksanen et al., 2008), with 999 permutations].

The association between environmental variables (geochemical measurements) and microbial community composition was evaluated with canonical analysis of principal coordinates (implemented in the vegan package) based on weighted UniFrac distances, followed by PERMANOVA to partition variance explained by soil geochemistry.

## Results

### Soil Chemical Analysis

**FIGURE 1:**
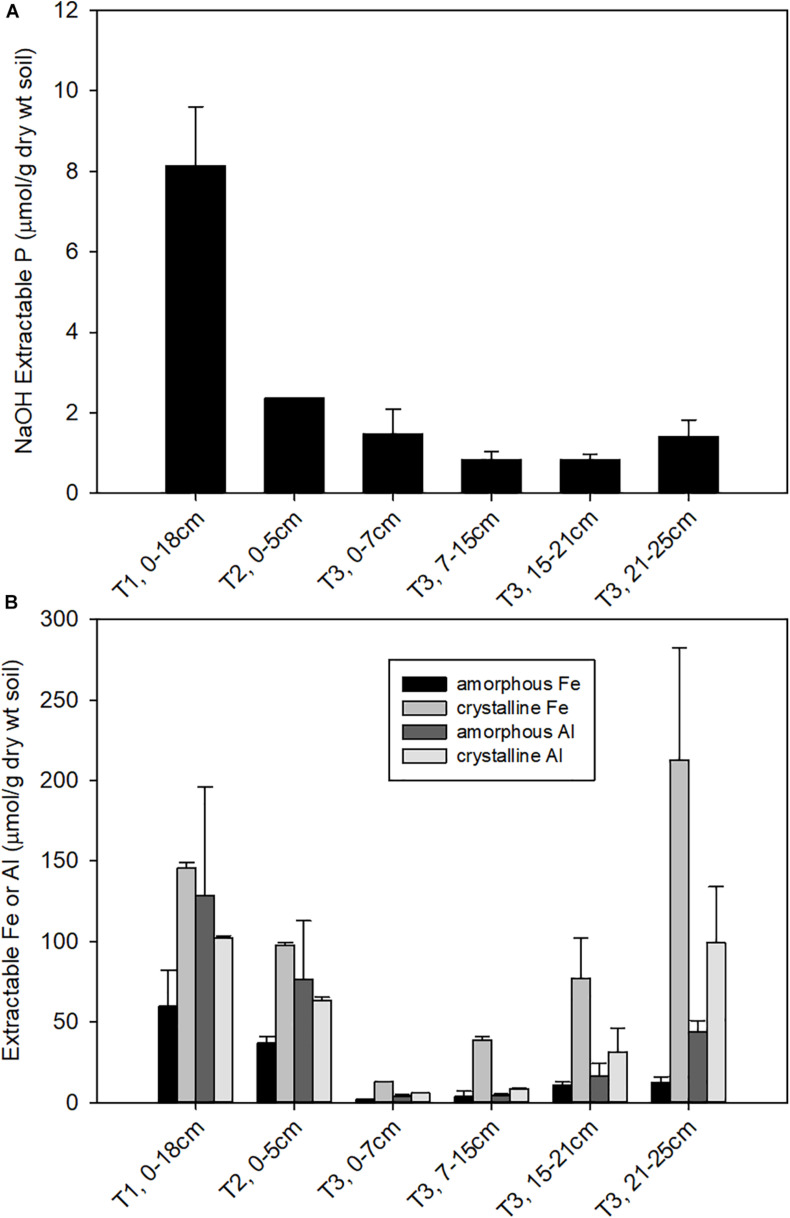
Concentrations of **(A)** NaOH-EDTA extractable P, representing organic and moderately bound inorganic P; and **(B)** oxalate and CBD extractable Fe, representing amorphous and total free (crystalline + amorphous) iron oxide contents, respectively.

The soil properties differed substantially across the three terraces. Surface soil pH (Supplementary Table 2) decreased from T1 (pH = 5.6 ± 1.2) to T2 (pH = 4.5 ± 0.5) to T3 (pH 3.6 ± 0.01), consistent with previous observations (Northup et al., 1995, 1998). The pH of T3 soils increased with depth to 4.3 below 15 cm. NaOH-EDTA extractable P decreased substantially from T1 to T2, with a further decline from T2 to T3. In T3, NaOH-EDTA extractable P decreased with soil section depth (Figure 1A). Soils were also characterized for extractable Fe and Al, where oxalate extractable metals represent a pool of poorly crystalline metal oxides and CBD extractable metals represent more crystalline metal oxides (Supplementary Table 2). Extractable Fe and Al decreased constantly from T1 to T2. Within T3, the deeper soils had higher extractable Fe and Al compared to the shallow soils (Figure 1B), where crystalline iron oxides become dominant. CEC also decreased from T1 to T3 (Supplementary Figure 1), with Al becoming the dominant exchangeable cation in T3 and micronutrients like Ca, K, Mg become less available from T1 to T3 as a result of weathering. Analysis of soil mineralogy identified iron and aluminum phosphate bearing minerals as the primary inorganic forms of P, while NMR analysis identified phytic acid as the dominant form of organic P in these acidic soils samples (Nico et al., unpublished data).

### Elemental Associations Analysis

**FIGURE 2:**
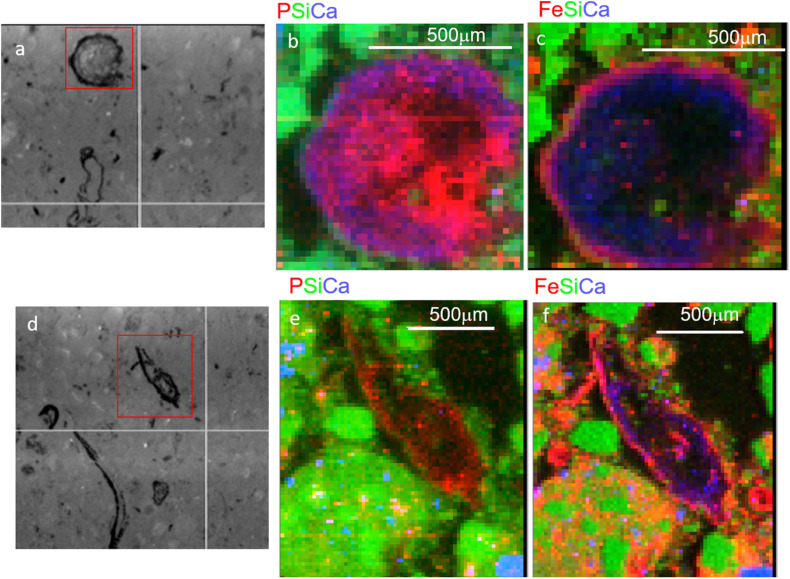
Micro x-ray fluorescence (μ-XRF) maps of soil from Terrace 3 at 15–21 cm depth. Two regions with organic-rich detritus are shown, with microscope images in panels **(a,d)**, and tri-color elemental maps (area indicated by red box) for P, Si, and Ca **(b,e)** and Fe, Si, and Ca **(c,f)**. Note the smaller red dots scattered throughout **(e)** are artifacts from the high Si concentrations and do not represent actual P.

We first used coarse maps to identify regions P rich regions in soil thin sections (P hot spots) and explored those regions using fine scale μ-XRF elemental maps. These elemental maps show the physical associations of P with other elements. In T1 soils, we observed a diversity of P associations in μ-XRF elemental maps, with P associated with both organic matter (e.g., litter fragments) and mineral phase. However, in T3, the hot spots of P observed were associated with organic matter (Figure 2). In some cases, P was concentrated in the center of the organic particle, with Fe present as a rind surrounding the particle (Figures 2b,c), while other spots showed a co-localization of P and Fe concentrated at the edges of the organic particles (Figures 2e,f). P and Fe were not co-localized in T1 and T2 (Supplementary Figure 2). These observations are consistent with a decrease in plant-available inorganic P from T1 to T3, and the predominance of organic P in T3, in some cases closely associated with Fe.

### Microbial Community Analysis

Overall, 5,142,729 sequences passed quality filtering; a range of 13,940–1088132 sequences obtained per soil sample. OTUs across the gradient were defined at 97% sequence homology and were distributed among 30 bacterial, 2 archaeal, and 1 unassigned division, with most the abundant belonging to *Proteobacteria* (42.6%), *Acidobacteria* (34.5%), *Verrucomicrobia* (8%), *Actinobacteria* (6.3%), *Planctomycetes* (3.2%) together representing 94.6% of all reads. The relative abundance of *Acidobacteria* steadily increased along the gradient (T1–T3) (*P* < 0.005). In contrast, a decrease was noted for *Verrucomicrobia*, *Bacteroidetes*, *Nitrospirae*, and *Gemmatimonadetes* with increasing terrace age (*P* < 0.05) along the gradient T1-T3.

**FIGURE 3:**
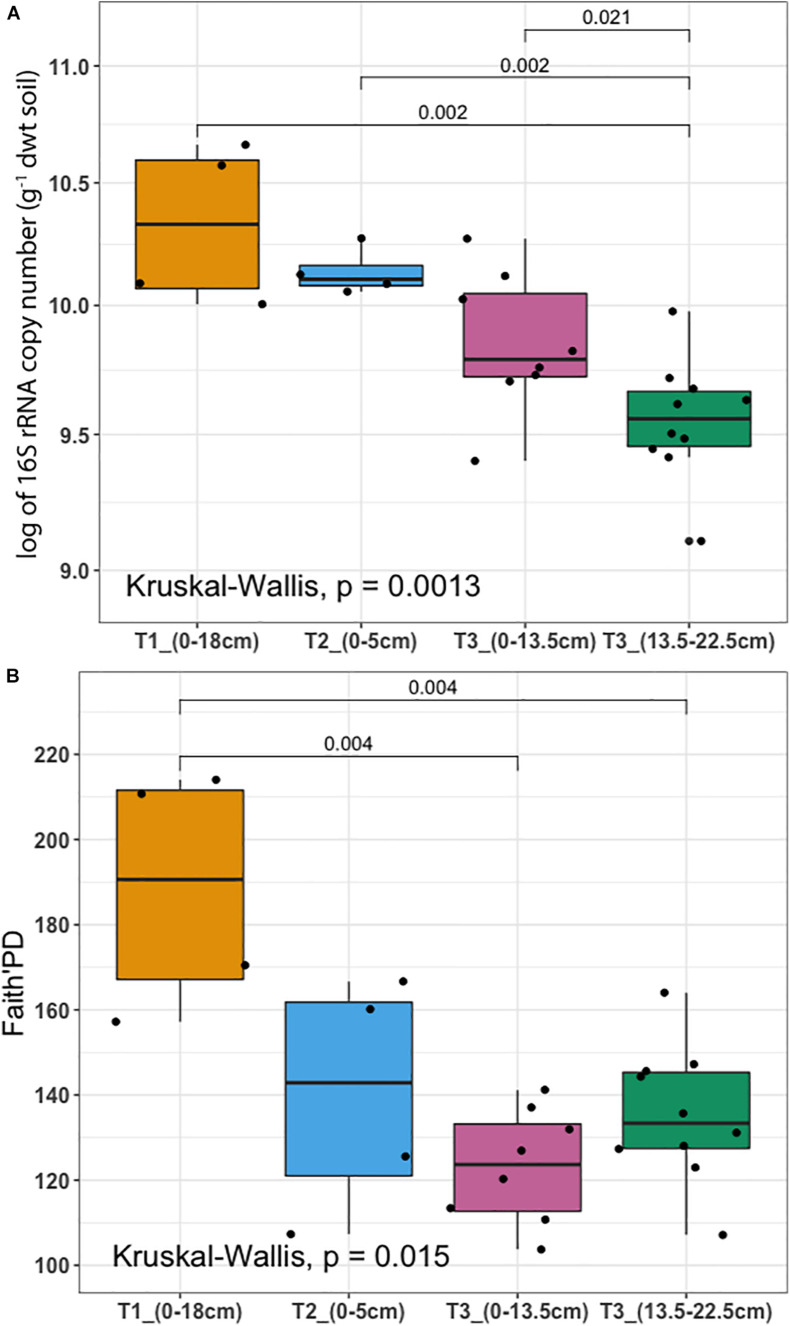
Microbial biomass and diversity analysis **(A)** 16S rRNA gene copy number per gram of dry weight soil assessed by qPCR (*y*-axis was logarithmically transformed). **(B)** Faith’s Phylogenetic Diversity (Faith’s PD). Samples were collected from three terraces with different depths: T1_(0–18 cm) (yellow), T2_(0–5 cm) (blue), T3_(0–13.5 cm) (pink), and T3_(13.5–22.5 cm) (dark green). In each box plot, a point denotes a single soil sample. The top and bottom of each box represent the 25th and 75th percentiles, the horizontal line inside each box represents the median and the whiskers represent the range of the points excluding outliers. Differences between terraces were evaluated using the Kruskal-Wallis analysis of variance, followed by *post hoc* Mann-Whitney-Wilcoxon test pairwise differences.

To determine whether microbial communities were structured differently across terraces, we analyzed a proxy for both bacterial/archaeal (hereafter termed microbial) biomass (based on qPCR of 16S rRNA genes/g soil) in addition to diversity (based on Faith’s PD). Microbial biomass was highest at T1 and decreased significantly toward T3 (*P* < 0.01) (Figure 3A). Diversity was highest at T1 and decreased across gradient, however, there was no significant difference between T2 and T3, or between different soil depths in T3 (Figure 3B). The soil microbiome differed in structure and relative abundance across the gradient, both horizontally (Adonis analysis: *F* = 6.2379, pseudo-R2 = 0.47387, *P* < 0.001) and vertically (*F* = 6.8921, pseudo-R2 = 0.52613, *P* < 0.001); T1 clearly separated from other soils in terms of microbial structure and abundance (Supplementary Figure 3A). While soils from T2 and T3 were also distinct, soils from T2 surface appeared more similar to the deeper soils from T3 (confidence level 0.95). Constrained ordination analysis showed the observed microbiome clustering was related to differences in a number of soil chemical properties including total extractable P, extractable iron content and other environmental variables (Supplementary Figure 3B).

**FIGURE 4:**
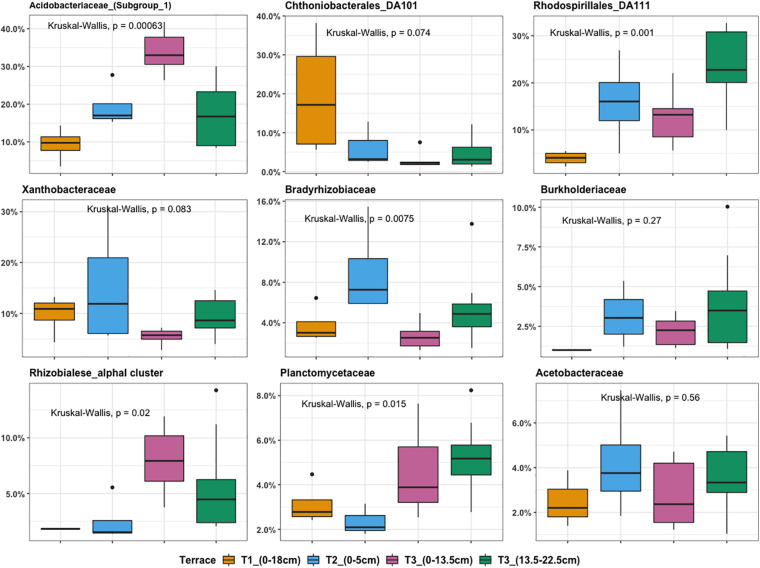
Boxplot illustrating the most abundant bacterial families ranked by relative OTU distribution across terraces. The top and bottom of each box represent the 25th and 75th percentiles, the horizontal line inside each box represents the median and the whiskers represent the range of the points excluding outliers. Outliers are denoted as large points outside whiskers. Significant contributions of terrace locations and depths to observed differences in OTU relative abundance were tested using Kruskal-Wallis test.

Microbial community composition analysis showed that there were in total, 224 bacterial families in the whole dataset. Taxonomic assignment indicated that the most abundant bacterial families were *Acidobacteriaceae* (Subgroup 1) (14.65%), *Rhodospirillales* DA111 (10.9%), *Xanthobacteraceae* (6.1%), and *Chthoniobacterales* DA101 soil group (4.2%). In general, *Chthoniobacterales* DA101 was the most abundant family in T1, while T2 was enriched in representatives from *Xanthobacteraceae*, *Acidobacteriaceae* (Subgroup 1), and *Rhodospirillales* DA111. The most representative families found in T3 were *Acidobacteriaceae* (Subgroup 1) and *Rhodospirillales* DA111. *Acidobacteriaceae* (Subgroup 1) and *Rhodospirillales* DA111 had an increasing trend across the gradient while *Chthoniobacterales* DA101 soil group decreased (Figure 4). Relative abundance of *Bradyrhizobiaceae* was highest at T2 and *Planctomycetaceae* was highest at T3. In agreement with the NMDS ordination results, T2 and deeper T3 (13.5–22.5 cm) showed similar patterns in relative abundance of most of the top families (Figure 4). Relative abundance of *Acidobacteriaceae* and *Rhizobiales* alpha I cluster were higher in T3 (0–13.5 cm) compared to T3 (13.5–22.5 cm). In contrast, *Rhodospirillales* DA111 increased along depths within T3. *Burkholderiaceae* were more abundant in deeper T3 (13.5–22.5 cm) (3.3%) and T2 (3.1%), compared to shallow T3 (0–13.5 cm) (1.7%) and T1 (0.5%).

### Isolation and Identification of Phosphate Solubilizing Bacteria From the Ecological Staircase

Hundreds of bacterial isolates were obtained from the Ecological Staircase soils (hereafter termed ES isolates) on modified NM8 medium with three different insoluble P sources: AlPO_4_, FePO_4_, and phytic acid. Ninety-six colonies based on different morphology recovered from each P source were selected for growth in liquid NM8 medium for identification and evaluation of P solubilization traits.

**FIGURE 5:**
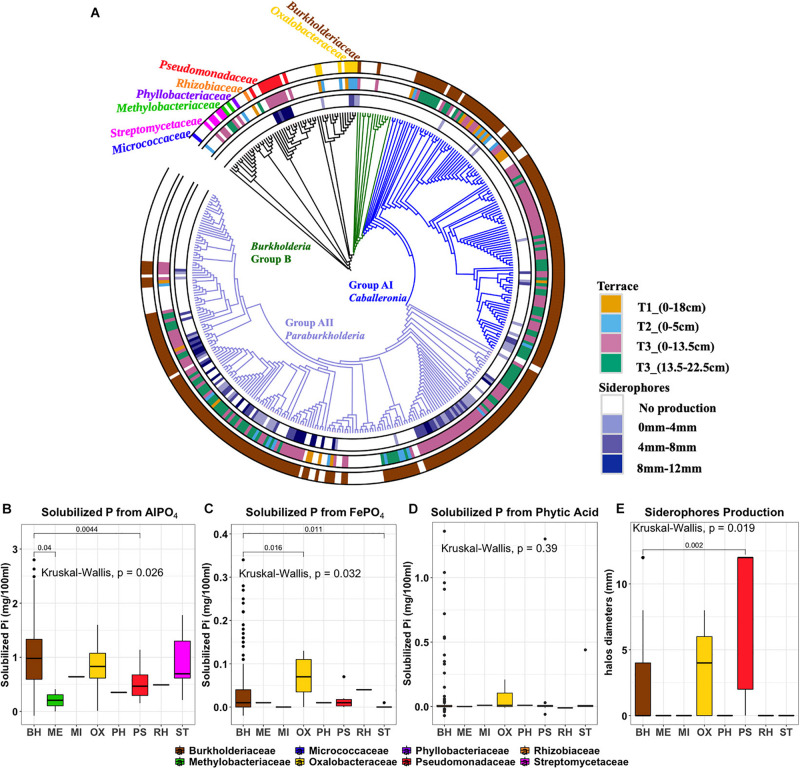
**(A)** Phylogenetic tree of 270 ES isolates and representative sequences as references. Rings, from the outer to the inner circles, represent: Ring1, taxonomy at family level. Ring2, Terrace of origin. Ring3: Siderophore production, with color gradient representing the diameter of colored halo production. **(B)** aluminum phosphate solubilization **(C)** iron phosphate solubilization **(D)** phytic acid solubilization **(E)** siderophore production. Outliers are denoted as large points outside whiskers. The Kruskal-Wallis test was used to compare the differences of P solubilization between isolates from different bacterial families. The Mann-Whitney-Wilcoxon was used to conduct pairwise *post hoc* comparisons.

The isolates obtained from the three insoluble P sources (*n* = 277) were taxonomically characterized by sequencing 16S rRNA gene loci (277 sequences of total 288 isolates passed QC). Full-length sequences identified the isolates as members of *Micrococcaceae* (*n* = 1), *Streptomycetaceae* (*n* = 5), *Methylobacteriaceae* (*n* = 2), *Phyllobacteriaceae* (*n* = 1), *Rhizobiaceae* (*n* = 1), *Pseudomonadaceae* (*n* = 9), *Oxalobacteraceae* (*n* = 7), and *Burkholderiaceae* (*n* = 251). Although the *Burkholderiaceae* were estimated by 16S iTag sequencing to be of lower abundance in the soil microbial community, they comprised the majority of recovered isolates (>90%). To link the isolates to the OTUs, both iTag and Sanger sequences were clustered at 100% sequence identity. Their relative abundances and phenotypic traits across the gradient are shown in Supplementary Figure 7. The *Burkholderiaceae* family separated into two distinct phylogenetic clades: *Burkholderia* Groups A and B (Figure 5A). Group A is phylogenetically diverse and consists of two branching 16S rRNA lineages: Group AI *Caballeronia* and Group AII *Paraburkholderia*. The first deep-branching Group AI *Caballeronia* comprised 104 ES isolates and a few reference species isolated from different ecological niches. The second deep-branching Group AII *Paraburkholderia* harbors 145 ES isolates and 58 primarily environmental and plant associated species as references.

### Phosphorus Solubilization and Siderophore Production of ES Isolates

Phenotypic traits of isolates including P solubilization and siderophore production were plotted according to their phylogenetic affiliation and annotated by geographic origin (terrace) and isolation origin (isolation medium) (Figure 5 and Supplementary Table 3). At the family level, the distributions of AlPO_4_ and FePO_4_ solubilization traits were significantly differences in different families (*P* < 0.05), where *Burkholderiaceae* isolates have a relative high AlPO_4_ and FePO_4_ solubilization (Figures 5B,C). No significant differences were found on phytic acid solubilization of different families (*P* = 0.39). However, most phytic acid solubilizers were *Burkholderiaceae* spp. (Figure 5D). Those *Burkholderiaceae* isolated from deeper T3 soils where the *Burkholderiaceae* were most abundant (13.5–22.5 cm) had highest phytic acid solubilization (*P* < 0.001) (Supplementary Figure 4A) compared to *Burkholderiaceae* isolates from other terraces. And the *Burkholderiaceae* isolates that produced more siderophores also showed much higher FePO_4_ solubilization (*P* ≤ 0.05) (Supplementary Figure 4B).

Siderophore production appeared as a distinctive phenotypic trait with clusters apparent among the different phylogenetic clades (Figure 5, ring 3). All isolates from the *Pseudomonadaceae* family showed strong siderophore production (Figure 5E). Within the *Burkholderiaceae* family, siderophores production was observed by 84 of 132 isolates identified as *Paraburkholderia* but only 14 of 100 isolates identified as *Caballeronia* within *Burkholderia* Group A.

Most ES isolates were able to utilize AlPO_4_ (277 isolates), with fewer subsisting on FePO_4_ (73 isolates) or the primary organic form, phytic acid (22 isolates) (Supplementary Figure 5). The amount of solubilized PO_4_^2–^ ranged between 0.3 and 28 μg/ml (representing 0.05–58.97% of the insoluble PO_4_^2–^ source). The range of insoluble P solubilized varied by P form: AlPO_4_ (0.63–58.97%), FePO_4_ (0.77–7.15%), and phytic acid (0.05–5.24%). A number of isolates (*n* = 82) showed the ability to solubilize more than one phosphorus forms and 11 of these isolates solubilized all three phosphorus forms: *Burkholderia* PA-D4, *Burkholderia* PA-B7, *Burkholderia* AL-E11, *Burkholderia* FE-C7, *Burkholderia* FE-E3, *Burkholderia* PA-C5, *Burkholderia* AL-F9, *Burkholderia* AL-D6, *Collimonas* AL-A1, *Collimonas* FE-F2, and *Pseudomonas* PA-B8. Siderophore production (identified by halo formation surrounding colonies) was identified in 39% of AlPO_4_ derived, 43% of FePO_4_ derived, and 37% of phytic acid derived isolates.

### Relationship Between Phenotype Traits and Isolation Media and Terrace Source of Isolate

**FIGURE 6:**
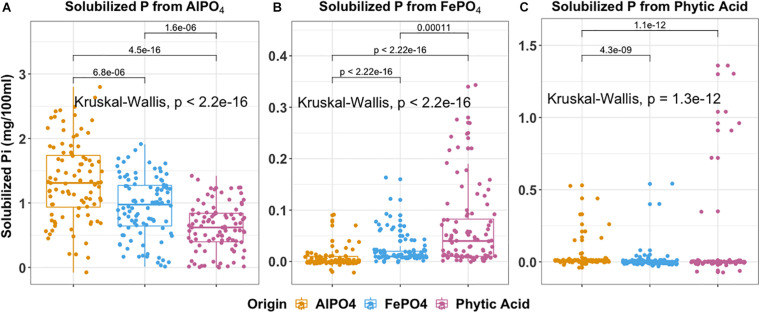
Boxplot indicates P solubilization ability of 288 isolates grouped by the isolation medium of origin. **(A)** aluminum phosphate solubilization **(B)** iron phosphate solubilization **(C)** phytic acid solubilization. In each box plot, a point denotes a single ES isolate. The top and bottom of each box represent the 25th and 75th percentiles, the horizontal line inside each box represents the median and the whiskers represent the range of the points excluding outliers. Kruskal-Wallis was used to compare the differences in P solubilization between bacteria obtained using different isolation media. The Mann-Whitney-Wilcoxon was used to conduct pairwise *post hoc* comparisons.

We specifically asked whether isolation medium could be related to P solubilization phenotype and present the findings in Figure 6. The ability to solubilize AlPO_4_ (*P* < 0.001), FePO_4_ (*P* < 0.001), and phytic acid (*P* < 0.001) significantly differed according to isolation medium. This produced some interesting observations. For example, isolates derived from AlPO_4_ medium were most effective at solubilizing AlPO_4_ (Figure 6A). Interestingly, isolates derived from phytic acid were most effective at solubilizing FePO_4_ (*P* < 0.01) (Figure 6B), and isolates derived from FePO_4_ were only more effective at solubilizing FePO_4_ than those derived from AlPO_4_ (*P* < 0.0001). Isolates derived from FePO_4_ and phytic acid were more effective at solubilizing phytic acid than isolates from AlPO_4_. However, no significant differences were found in phytic acid solubilization between isolates derived from FePO_4_ and phytic acid. This suggests that organisms adapted to solubilize phytic acid are also highly effective at solubilizing FePO_4_ and suggests that Fe solubilization and organic P hydrolysis traits may be linked. Related to this FePO_4_ solubilization capacity significantly differed across different siderophore production groups (*F* = 5.469, *P* ≤ 0.001). Strong siderophores producers (siderophore halos > 8 mm) displayed much higher FePO_4_ solubilization than those with no siderophore production (8–12 mm vs. 0, *P* < 0.05).

The distributions of P solubilization traits and siderophores production traits across gradient were summarized in Supplementary Figure 6. The distributions of phytic acid solubilization traits were (*P* < 0.001) significantly different across gradient (Supplementary Figure 6C). T3_(13.5–22.5 cm) harbors the most isolates with the highest phytic acid solubilization compared to other terraces (*P* < 0.05). No significant differences in the distributions of individual AlPO_4_ and FePO_4_ solubilization and siderophores production traits were observed across the gradient (AlPO_4_: *P* = 0.15; FePO_4_: *P* = 0.2; Siderophores: *P* = 0.19). However, the majority of organisms with a high potential for FePO_4_ solubilization and siderophores production originated from T3 (Supplementary Figures 6B,D).

### Evaluating the Influence of Iron Complexation/Co-precipitation on Enzyme (Phytase) Hydrolysis and Release of P From Phytic Acid

**FIGURE 7:**
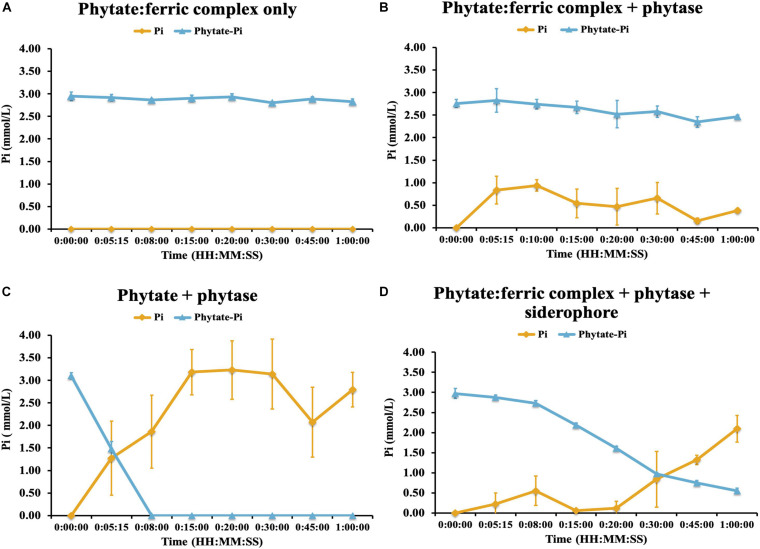
Phytase hydrolysis kinetics on ferric-complexed phytate: **(A)** ferric-phytate complex only **(B)** ferric-phytate complex incubated with phytase **(C)** phytate incubated with phytase **(D)** ferric-phytate complex incubated with phytase and a siderophore (ferrichrome). Concentration of free PO_4_ (yellow line) and PO_4_ of phytate (blue line) were measured by ion chromatography over 1 h. Each treatment had three replicates. Standard deviations are indicated by the error bars.

We next evaluated the potential control by Fe-complexation on phytase-mediated inorganic P (Pi) release from phytic acid and the potential for the linked traits of siderophore and phytase production to alleviate those constraints. To do this we performed an experiment where we created an iron-phytate complex and evaluated the ability of phytases to hydrolyze iron complexed organic P in the presence and absence of a bacterial hydroxymate siderophore (ferrichrome). This experiment demonstrated that no additional Pi was liberated by phytase when phytate was complexed with iron (Figure 7). Next we evaluated the ability of the siderophore to chelate Fe from the phytate:ferric complex and enable phytase-mediated dephosphorylation of phytic acid. This demonstrated that Pi was liberated from Fe complexed phytic acid only after adding the siderophore (Figure 7), confirming that siderophore-mediated chelation of Fe enables phytase access to phytic acid and Pi solubilization. This constraint imparted by Fe complexation of organic P may be an important regulator of soil P availability although it appears that a small subset of soil bacteria (in this case) can access this form of chemically recalcitrant P due to their linked traits of siderophore and phytase production.

## Discussion

In this study, we set out to evaluate whether the preponderance of occluded and insoluble P selects for microorganisms with specific traits, the phylogenetic distribution of these traits within and between bacteria, and how these traits might be associated with P mobilization *in situ*. A critical consideration in assessing microbial phenotypic traits associated with P solubilization involves the design of media with P forms representative of the conditions to which the microorganisms have adapted. The predominant forms of insoluble P vary across soils, with calcium phosphates (Ca-Pi) dominating above pH 6 and aluminum and iron phosphates (Al-Pi and Fe-Pi) below pH 5.5 (Childers et al., 2011). Despite the global bimodal distribution of soil pH (Slessarev et al., 2016), most studies have selected Ca-Pi as the sole P source for cultivating P solubilizing microorganisms (e.g., Illmer and Schinner, 1992; Chen et al., 2006; Oliveira et al., 2009; Park et al., 2010; Uroz et al., 2014; Wei et al., 2018), which may not be as relevant to *in situ* conditions. High growth rates of microorganisms on laboratory media are often associated with proton release and hence dissolution of Ca-Pi. Such growth-related proton release, however, is not effective in mobilizing P from Fe-Pi or Al-Pi or Pi adsorbed to Fe or Al oxides (Jones and Oburger, 2011), which requires the secretion of organic ligands such as organic acid anions or siderophores. Hence, the capacity to mobilize Pi from Fe-Pi or Al-Pi is expected to be less common, and Pi mobilization rates are often lower than those for Ca-Pi (Banik and Dey, 1982). Under nutrient-deficient conditions, and hence lower microbial growth rates, P solubilization is often strongly diminished (Marschner, 2008) and isolates selected as strong P solubilizers by using Ca-Pi *in vitro* may not be as relevant to *in situ* conditions (Gyaneshwar et al., 2002; Rengel and Marschner, 2005; Bashan et al., 2013). The selection of the inorganic P species for potential PSB should depend on the type of soil (alkaline, acidic, or organic-rich) where the PSB inhabit or will be used.

In this study, cultivation media were designed using carbohydrates typical of root exudates (to cultivate microorganisms that might be expected to inhabit the rhizosphere) together with insoluble P forms relevant to the acidic and highly weathered soils of this location. This yielded hundreds of P-mobilizing and siderophore-producing microorganisms, with a significant representation of *Burkholderiaceae* (*Caballeronia* and *Paraburkholderia* spp.) in addition to *Pseudomonas*, *Methylobacter*, *Rhizobium*, and *Collimonas* spp. amongst others. The *Burkholderiaceae* family was also observed via iTag sequencing to increase in relative abundance across the terraces in parallel with P limitation. We observed that the extent of P solubilization from different insoluble P forms by the isolated microorganisms followed the order: AlPO_4_ > FePO_4_ > phytate, with almost all isolates capable of solubilizing AlPO_4_ but fewer microbes capable of solubilizing the organic form.

Evidence of Fe-complexation of P *in situ* came from μ-XRF elemental maps showing the physical association of P with other elements (Supplementary Figure 2). These combined with soil chemical analyses, suggest that across the staircase, as soils were increasingly weathered, P becomes increasingly dominated by organic forms and those organic-associated P hotspots are associated with an iron coating (Figure 2). Terrace 3 along the gradient harbored the most isolates with the highest phytate solubilization potential compared to other terraces. We also found that the P form used for isolation (AlPO_4_, FePO_4_, and phytic acid) influenced P solubilization capacity. A particularly intriguing finding was that bacteria isolated on phytate had the greatest potential to solubilize P from FePO_4_ suggesting that the adaptation to solubilize organic P in this system was linked to traits related to solubilizing Fe. In fact, the ability to produce siderophores was widespread amongst those bacteria that showed the highest P solubilization activity, again suggesting that Fe-complexation might be an important constraint on P availability.

Organic P can comprise up to 95% of total soil P, of which phytate is usually a major component (Dalai, 1977; Turner et al., 2002). Why phytate persists in soils when plants and microorganisms possess the enzymatic machinery to hydrolyze it has been subject to debate (see Gerke, 2015 and references therein). Phytate is known to bind strongly to Al-OH or Fe-OH-groups, leading to fixation by soil minerals, often more so than orthophosphate (McKercher and Anderson, 1989; Turner et al., 2002; Celi and Barberis, 2005), and this may explain its persistence as an abundant form of organic P in soils. Low extracellular phosphatase activity of plant roots has been invoked to explain phytate persistence in soil (Richardson, 2001), however the mobility of, and enzyme access to, metal-complexed phytate may be an explanation. In fact an anecdotal observation in our media preparation led us to follow this further. We noted that when phytate was added to unbuffered cultivation media containing FeOH that an insoluble precipitate formed immediately. We re-created this observation to evaluate the impact of this co-precipitation of phytate with FeCl_3_ on enzymatic hydrolysis of phytate by phytase and to determine if siderophores combined with phytase would enable access for plants and microorganisms to this typically recalcitrant pool. These experiments confirmed that the insoluble Fe-phytate precipitate was inaccessible to the phytase enzyme as noted previously for other systems (Dao, 2003; He et al., 2006). Importantly, we also confirmed that siderophore addition restored enzymatic access to phytate within insoluble Fe-phytate precipitates. Therefore, for microorganisms in acidic highly weathered soils, or even mildly acidic soils with abundant iron oxides, these linked traits would appear to be highly advantageous for P acquisition. There is some precedence for this. Observations in marine bacteria (e.g., *Pseudovibrio* sp. FO-BEG1) show that limiting Pi induced the expression of iron chelating compounds (Romano et al., 2017), hypothesized to facilitate access to Fe-adsorbed Pi (Ghosh et al., 2015). If this is a fundamental constraint on organic P availability in soils, then one would expect this pattern of trait-linkage to be widespread, at least for those organisms adapted to life in highly weathered soils.

The *Burkholderiaceae* comprises an incredibly diverse and versatile gram-negative family of bacteria spanning a range of human, animal, plant pathogens, as well as numerous genera with significant beneficial biotechnological potential associated with plants (Estrada-de los Santos et al., 2016; Kaur et al., 2017). Our phylogenetic analysis of the 16S rRNA gene sequences from 328 *Burkholderiaceae* (83 reference sequences and 243 ES isolates), identified two major clades similar to those described previously (Gyaneshwar et al., 2011; Sawana et al., 2014; Estrada-de los Santos et al., 2016). These two monophyletic groups A and B, consists of the genera *Caballeronia* and *Paraburkholderia* in group A, with most of the notable human, animal and plant pathogens (including the “*Burkholderia cepacia* complex”) in group B. In our study, 242/243 ES *Burkholderiaceae* isolates map to group A, a much larger group of species comprising plant-associated beneficial and environmental species that are primarily known not to be pathogenic (Calvaruso et al., 2006; Caballero-Mellado et al., 2007; Zakaria et al., 2007; Onofre-Lemus et al., 2009; Depoorter et al., 2016; Ghosh et al., 2016). The most effective P solubilizing microbial isolates we recovered belonged to the *Caballeronia* and *Paraburkholderia* genera (Irawan et al., 2020). Higher siderophore production in general (Figure 5), together with higher maximal values of phytate solubilization were observed in *Burkholderiaceae* isolates derived from T3 soils where Fe-complexed organic P appears to dominate (Supplementary Figure 4A). Together these data suggest that a subset of the *Burkholderiaceae* possess these linked traits to access the insoluble organic P pool. Ongoing analysis of their genomes will provide further insight into the extent of linkage and co-evolution of these important metabolic traits, as well as analysis of the co-occurrence of these traits in microorganisms that have yet to be cultivated.

## Conclusion

Over all we show that across a natural nutrient limitation gradient resident microorganisms possess distinct P solubilization strategies. In particular we show that the ability to solubilize P from AlPO_4_ appears to be widespread while the capacity to access P from FePO_4_ or phytate is less common. Our spectroscopic evidence for Fe-association with organic-matter associated P, coupled with *in vitro* experiments shows how Fe-complexation inhibits enzyme access to one of the most abundant organic P forms in soil. We show that the metabolic traits required to solubilize P from Fe-complexed phytate are restricted to a limited subset of the soil bacteria evaluated here. If the co-occurrence of these traits is similarly restricted across other soil bacteria, this may help explain why phytate persists in acidic soils.

## Data Availability Statement

The datasets presented in this study can be found in online repositories. The names of the repository/repositories and accession number(s) can be found below: NCBI BioProject, accession no: PRJNA643303.

## Author Contributions

SW, RC, PN, and EB designed the study, which is a contribution to a Laboratory Directed Research and Development (LDRD) project lead by EB and PN. SW, RW, MS, UK, PF, PN, RC, and EB performed the data collection and analysis. SW and EB wrote the manuscript. All authors contributed to revisions.

## Conflict of Interest

MS was employed by the company Pendulum Therapeutics after the conclusion of this work. The remaining authors declare that the research was conducted in the absence of any commercial or financial relationships that could be construed as a potential conflict of interest.

## Footnotes

1 http://drive5.com/uchime/gold.fa
