# Supplementary material for: Microbial Phosphorus Mobilization Strategies Across a Natural Nutrient Limitation Gradient and Evidence for Linkage With Iron Solubilization Traits
Source: Front Microbiol. 2021 Jun 23;12:572212. doi: 10.3389/fmicb.2021.572212 (PMC8261140; doi:10.3389/fmicb.2021.572212)
Supplement: Supplementary file 1 [file Data_Sheet_1.docx]

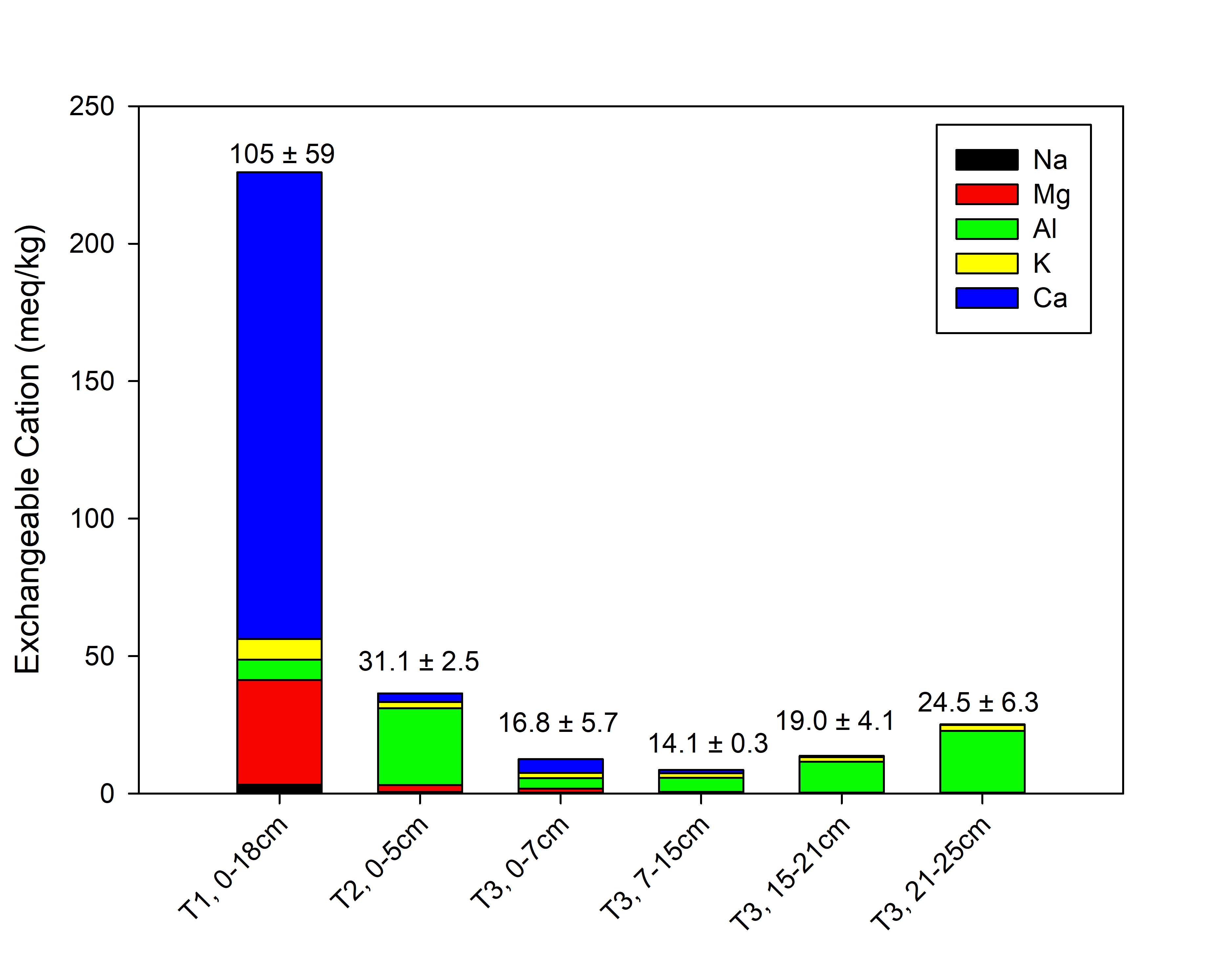


**Supplementary Figure 1.** Exchangeable cations for each soil. The total cation exchange capacity for each soil is indicated above the bars.


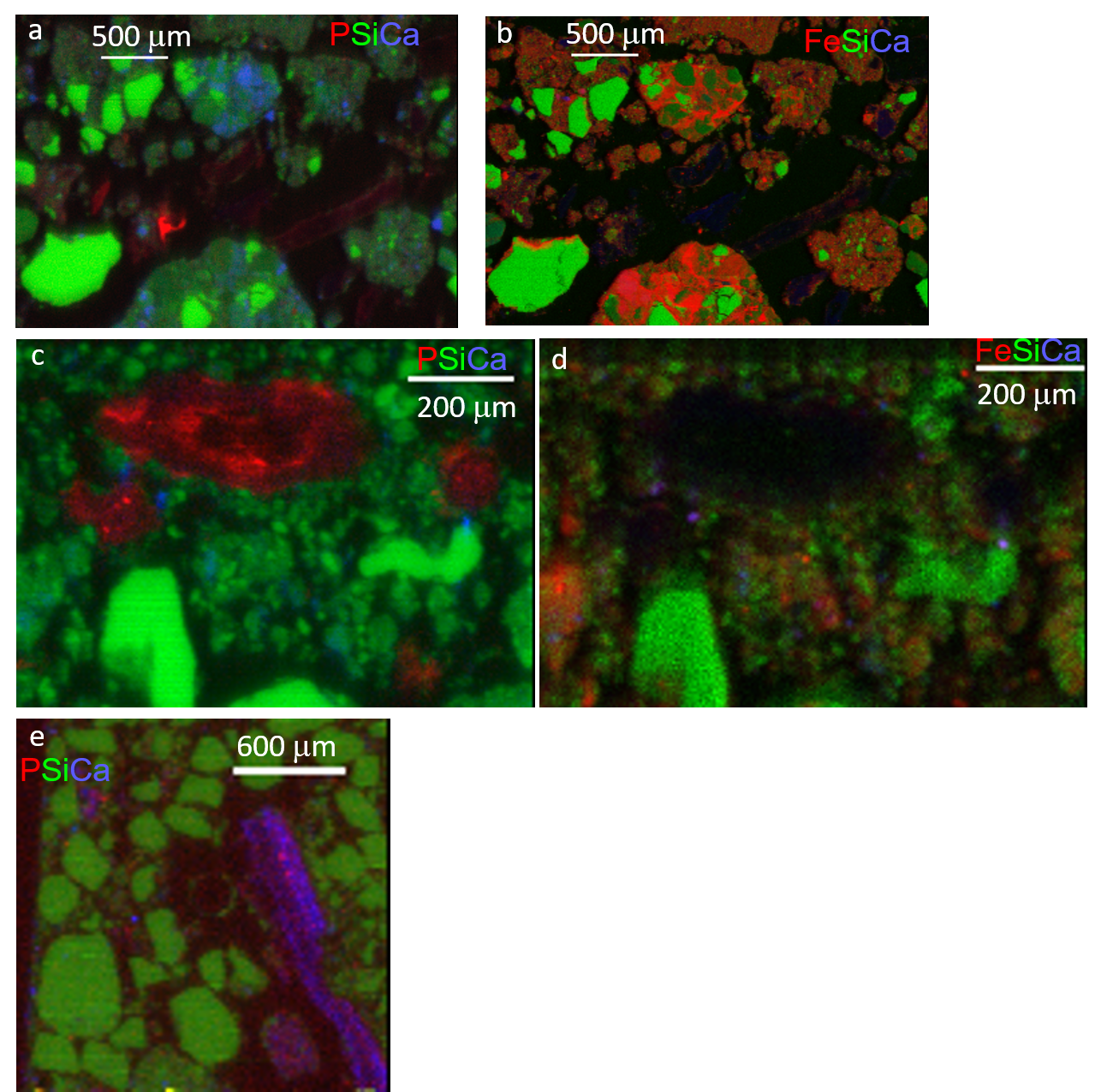


**Supplementary Figure 2.** Example µ-XRF maps of surface soil from (a, b) Terrace 1, 0-18 cm, (c-d) Terrace 2, 0-5 cm. (e) Terrace 3, 0-7 cm depth. Tri-color elemental maps show elemental associations for P, Si, and Ca (a, c) and Fe, Si, and Ca (b, d).

**
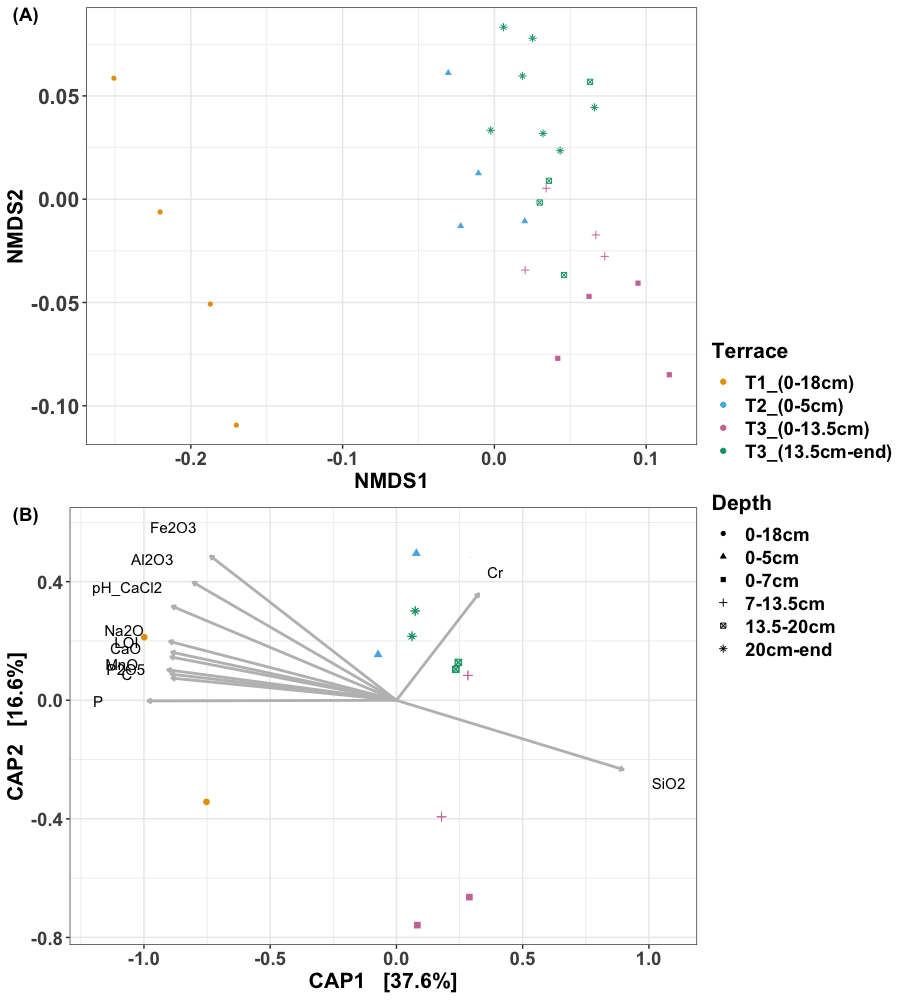
**

**Supplementary Figure 3.** **(A)** 16S rRNA gene iTag sequencing reveals the differences in microbiome composition of 27 soil samples from three different terraces and depths. Non-metric multidimensional scaling was based on weighted UniFrac distances. Significant contributions of different terraces and depths to observed differences in microbial composition were tested using ANOVA. **(B)** Constrained ordination analysis of proximities was used to visualize environmental variables (ALS mineral analysis) associated with changes in microbial community composition. The ordination axes were constrained to linear combinations of environmental variables. Permutational multivariate analysis of variance (Adonis) tests were performed on weighted UniFrac distances. Each point corresponds to a soil sample colored in yellow for T1_(0-18 cm), blue for T2_(0-5 cm), and pink for T3_(0-13.5 cm) and green for T3_(13.5 cm-end) respectively. The shapes of the points correspond to soil sample depths. The length of cores varies from 22.5cm to 25cm.


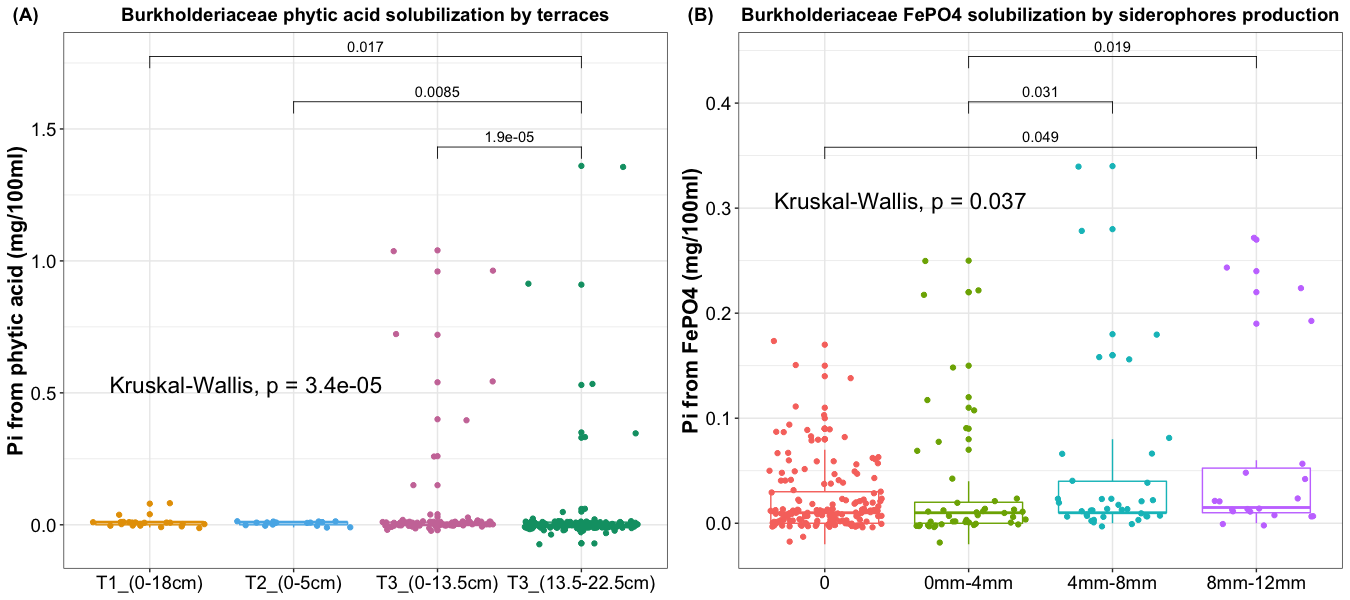


**Supplementary Figure 4**. **(A)** Boxplot of Pi concentrations liberated from phytic acid by *Burkholderiaceae* from different terraces. **(B)** Boxplot of Pi concentrations liberated from FePO_4_ by *Burkholderiaceae* isolates from different siderophores production groups. Vertical bars represent standard deviations of the mean. Top and lower lines of boxes represent, respectively, 75th and 25th percentiles. Horizontal bars represent the medians. Points outside the boxes represent outliers.

**
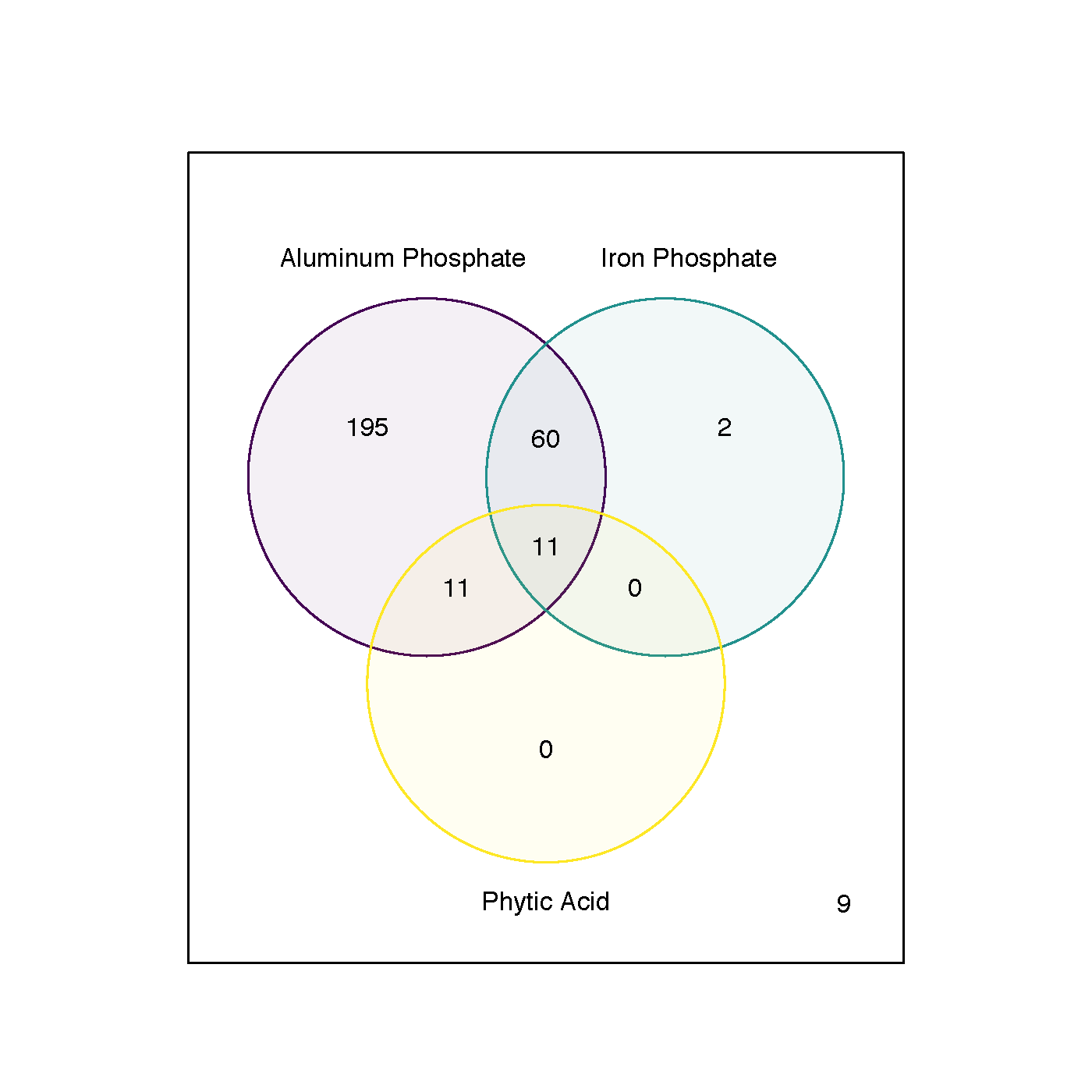
**

**Supplementary Figure 5.** Venn diagrams shows the intersection of bacterial isolate clusters and their P solubilization capacity.


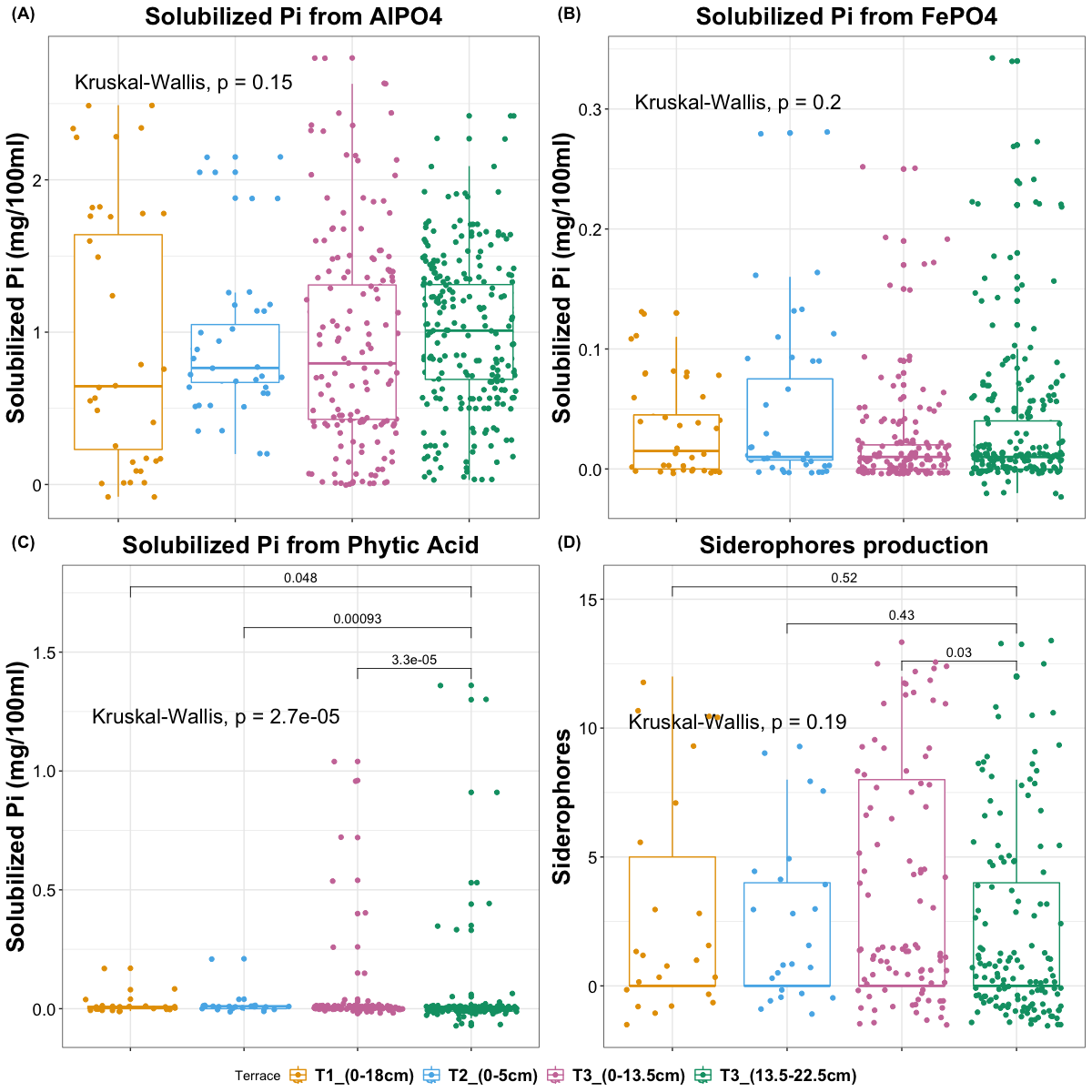


**Supplementary Figure 6.** (A) Boxplot of Pi concentrations liberated from AlPO_4_ by ES isolates derived from different terraces. (B) Boxplot of Pi concentrations liberated from FePO_4_ by ES isolates derived from different terraces. (C) Boxplot of Pi concentrations liberated from phytic acid by ES isolates derived from different terraces. (D) Boxplot of siderophores production by ES isolates derived from different terraces. Vertical bars represent standard deviations of the mean. Top and lower lines of boxes represent, respectively, 75th and 25th percentiles. Horizontal bars represent the medians. Points outside the boxes represent outliers.

**
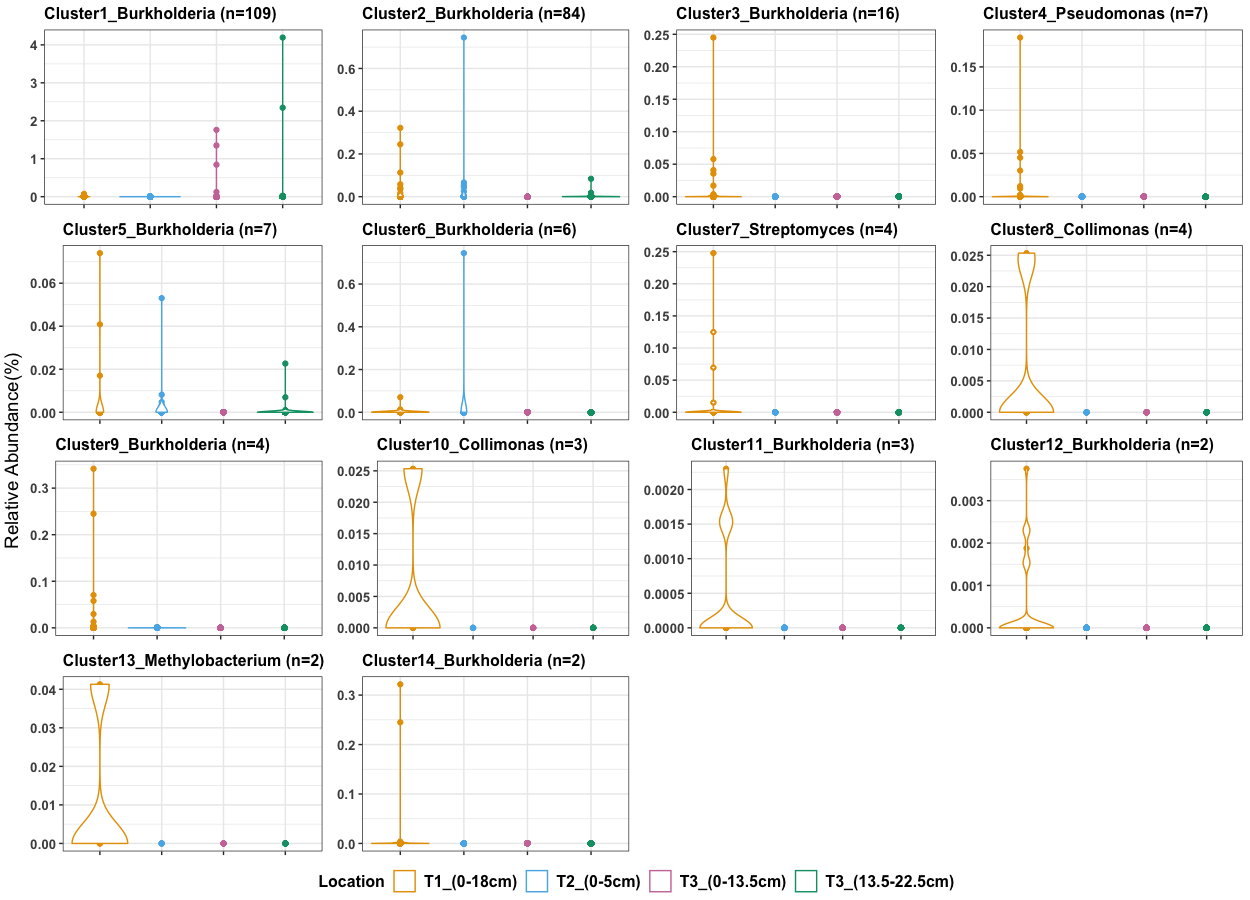

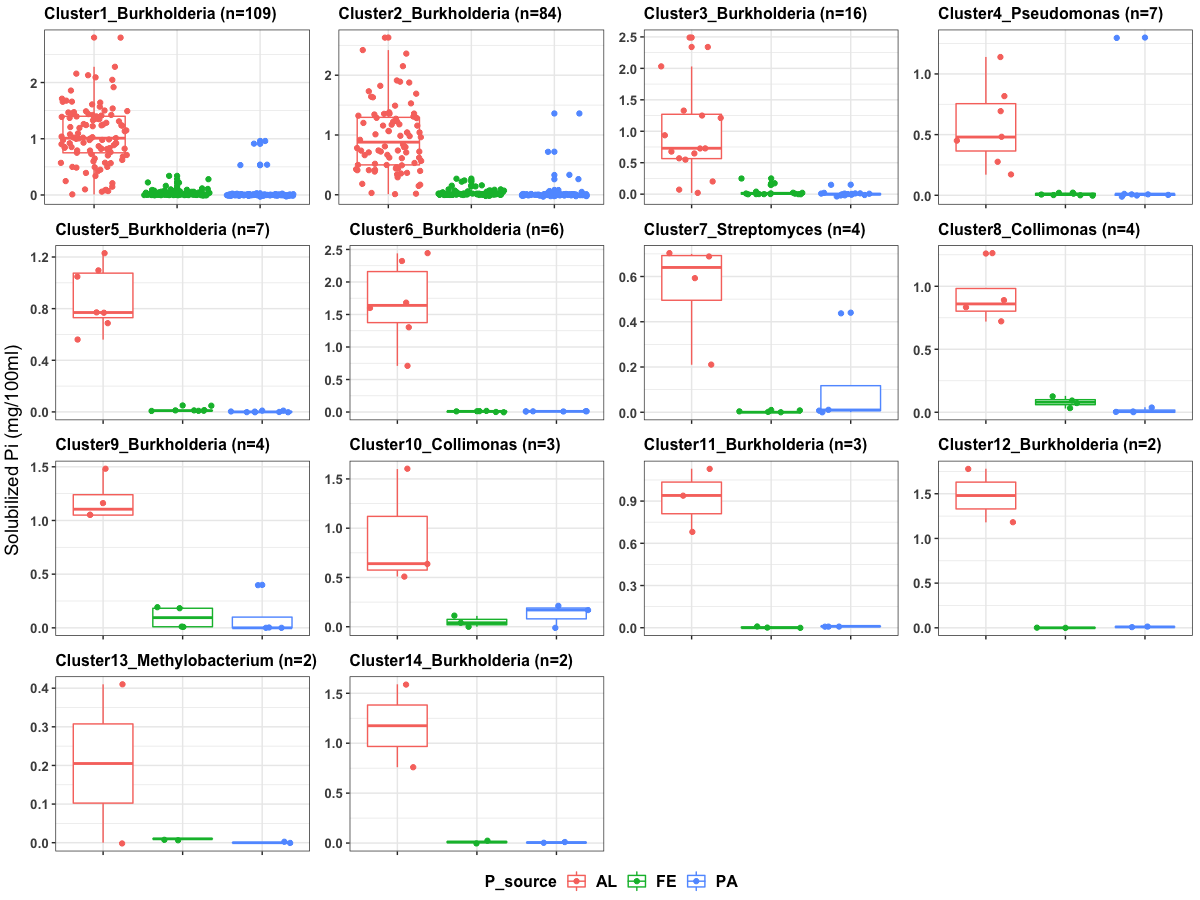
**

(A)

(B)

**Supplementary figure 7:** To link the isolates to the OTUs, for each isolate cluster, the OTUs with an identical sequence were determined. There are total 14 isolate clusters with number of isolates greater than or equal to two. Their relative abundances and phenotypic traits across the gradient were shown. (A) Point plot indicates the relative abundance of isolate clusters. (B) Boxplot indicates P solubilization by different isolate clusters.

|  | **GPS coordinate** | **Landscape** | **Vegetation** | **Age (years)** | **Total P^a^** | | **Horizon** | | **Depth^a^ (cm)** | | **N (g/kg)** | | **C (g/kg)** |  |
| --- | --- | --- | --- | --- | --- | --- | --- | --- | --- | --- | --- | --- | --- | --- |
| T1 | 39°22'31''N 123°49'11'' W | Coastal grassland | Grass | 100,000 | 194.58 ± 11.01 | O | | 8 | | 10 | | 431 | | |
|  |  |  |  |  |  | A | | (26) 20 | | 2.2 | | 36.1 | | |
| T2 | 39°22'30''N 123°47'48'' W | Forest | Bishop pine (*Pinus muricata*) | 200,000 | 133.27 ± 9.32 | O | | (11) 10 | | 8.7 | | 362 | | |
|  |  |  |  |  |  | A | | (12) 13 | | 1.5 | | 33.1 | | |
| T3 | 39°22'28''N 123°47'19'' W | Pygmy forest | Mendocino cypress (*Cupressus pygmaea*), | 240,000 | 127.87 ± 13.94 | O | | (4) 4 | | 5.8 | | 378 | | |
|  |  |  |  |  |  | A | | (4) 3 | | 1.8 | | 70.5 | | |
|  |  |  |  |  |  | E1 | | (4) 7 | | 0.8 | | 29 | | |
|  |  |  |  |  |  | E2 | | (18) 18 | | 0.7 | | 4.3 | | |
|  |  |  |  |  |  | Bh | | (14) 12 | | 1 | | 21.4 | | |

**Supplementary Table 1.** Selected terrace characterization data for three terraces along the Ecological Staircase chronosequence (extracted from Northup et al. 1998 and Izquierdo et al. 1999).

^a^ P fractions and Total P pools (g/m^2^) to a depth of 40 cm across three terraces. Values are weighted means across horizons ± 1 SE. All P fractions were calculated using the modified Hedley fractionation procedure

**Supplementary Table S2.** Soil chemical characteristics, including pH, oxalate and CBD-extractable Al and Mn, NaOH-EDTA extractable P, and total Al, Fe, and P.

| **Terrace,**  **Depth** |  |  | **Oxalate^a^** | | **CBD^b^** | | **NaOH** | **Total** | | |
| --- | --- | --- | --- | --- | --- | --- | --- | --- | --- | --- |
|  | **pH_w_** | **pH_Ca_** | **Al** | **Mn** | **Al** | **Mn** | **P** | **Al** | **Fe** | **P** |
|  |  |  | mmol/kg | | | | | | | |
| **Terrace 1** | | | | | | | | | | |
| 0-18 cm | 5.6 ± 1.2 | 5.3 ± 1.2 | 129 ± 67 | 11.27 ± 8.22 | 102 ± 1 | 11.00 ± 0.42 | 8.13 ± 1.47 | 918 ± 318 | 196 ± 90 | 17 ± 12 |
| **Terrace 2** | | | | | | | | | | |
| 0-5 cm | 4.5 ± 0.5 | 3.8 ± 0.0 | 76.5 ± 36.3 | 0.44 ± 0.41 | 63.4 ± 1.9 | 0.42 ± 0.02 | 2.36 ± 0.01 | 545 ± 70 | 113 ± 7 | 2.1 ± 1.0 |
| **Terrace 3** | | | | | | | | | | |
| 0-7 cm | 3.6 ± 0.0 | 3.3 ± 0.0 | 3.84 ± 0.82 | 0.036 ± 0.033 | 5.82 ± 0.01 | 0.040 ± 0.000 | 1.47 ± 0.62 | 87 ± 6 | 39 ± 0 | 1.4 ± 0.1 |
| 7-15 cm | 3.8 ± 0.1 | 3.4 ± 0.1 | 4.36 ± 0.87 | 0.013 ± 0.003 | 8.54 ± 0.20 | 0.023 ± 0.000 | 0.84 ± 0.20 | 98 ± 10 | 58 ± 18 | 1.4 ± 0.1 |
| 15-21 cm | 4.3 ± 0.2 | 3.7 ± 0.3 | 16.1 ± 8.0 | 0.011 ± 0.001 | 23.6 ± 0.1 | 0.028 ± 0.018 | 0.79 ± 0.19 | 166 ± 31 | 75 ± 15 | <1.4 |
| 21-25 cm | 4.3 ± 0.7 | 3.8 ± 0.4 | 43.8 ± 7.1 | 0.013 ± 0.001 | 60.3 ± 1.0 | 0.030 ± 0.000 | 1.23 ± 0.42 | 471 ± 219 | 128 ± 49 | 1.4 ± 0.1 |

pH_w_ = pH of soil in water
pH_Ca_ = pH of soil in 0.1 M CaCl_2_
^a^ Oxalate-extractable metals represents amorphous metal-oxide content.
^b^ CBD-extractable metals represents crystalline metal-oxide content.

**Supplementary Table S3.** ES isolates metadata (n=288) including taxonomy at family level, terrace of origin, P solubilization ability indicated by the concentration of solubilized phosphate and siderophores production indicated by halo size.

^a^ PA,AL,FE in strain ID represents the medium of isolate origins: phytic acid, aluminum phosphate and iron phosphate respectively.

^b^ ES Isolates were taxonomically characterized by sequencing 16S rRNA gene loci. N.A represents failed sequencing reactions.

^c^ Siderophores production measured by the diameter of halo on overlay CAS plate. 0 represents no halo. 1 represents the diameter of halo >0mm <=4mm. 2 represents the diameter of halo >4mm <=8mm. 3 represents the diameter of halo >8mm <=12mm.

|  | |  | |  | | **PO_4_^2-^ mg/100ml** | | | | | | |  | | |
| --- | --- | --- | --- | --- | --- | --- | --- | --- | --- | --- | --- | --- | --- | --- | --- |
| **Strain ID**^a^ | | **Family**^b^ | | **Terrace** | | **PA** | | **Fe-P** | **Al-P** | | **O-CAS halo**^c^ | | |  |  |
| PA-A1 | Oxalobacteraceae | | T1 0-18cm | | -0.01 | | 0.13 | | | 0.01 | | 0 | | |  |
| PA-B1 | Burkholderiaceae | | T1.3 | | -0.01 | | 0.11 | | | 0.64 | | 0 | | |  |
| PA-C1 | Burkholderiaceae | | T1.3 | | 0.00 | | 0.06 | | | 0.57 | | 3 | | |  |
| PA-D1 | Burkholderiaceae | | T1.3 | | -0.01 | | 0.01 | | | 0.55 | | 0 | | |  |
| PA-E1 | Burkholderiaceae | | T1.3 | | 0.00 | | 0.08 | | | 0.09 | | 0 | | |  |
| PA-F1 | Burkholderiaceae | | T1.3 | | 0.00 | | 0.03 | | | 0.15 | | 0 | | |  |
| PA-G1 | Rhizobiaceae | | T1.3 | | -0.01 | | 0.04 | | | 0.49 | | 0 | | |  |
| PA-H1 | N.A | | T1.3 | | -0.01 | | 0.08 | | | 0.25 | | 0 | | |  |
| PA-A2 | Burkholderiaceae | | T2.3 | | 0.00 | | 0.16 | | | 0.52 | | 2 | | |  |
| PA-B2 | Burkholderiaceae | | T2.3 | | -0.01 | | 0.28 | | | 1.00 | | 2 | | |  |
| PA-C2 | Oxalobacteraceae | | T2.3 | | 0.00 | | 0.07 | | | 0.83 | | 2 | | |  |
| PA-D2 | Burkholderiaceae | | T2.3 | | 0.00 | | 0.11 | | | 0.60 | | 1 | | |  |
| PA-E2 | Burkholderiaceae | | T2.3 | | -0.01 | | 0.09 | | | 0.70 | | 1 | | |  |
| PA-F2 | Burkholderiaceae | | T2.3 | | 0.00 | | 0.05 | | | 0.77 | | 0 | | |  |
| PA-G2 | Oxalobacteraceae | | T2.3 | | 0.01 | | 0.03 | | | 0.72 | | 2 | | |  |
| PA-H2 | Oxalobacteraceae | | T2.3 | | 0.00 | | 0.13 | | | 0.89 | | 0 | | |  |
| PA-A3 | Burkholderiaceae | | T3.2 0-7cm | | -0.02 | | 0.15 | | | 0.55 | | 0 | | |  |
| PA-B3 | Burkholderiaceae | | T3.2 0-7cm | | 0.00 | | 0.07 | | | 0.49 | | 1 | | |  |
| PA-C3 | Burkholderiaceae | | T3.2 0-7cm | | 0.00 | | 0.02 | | | 0.40 | | 1 | | |  |
| PA-D3 | Burkholderiaceae | | T3.2 0-7cm | | 0.00 | | 0.09 | | | 0.06 | | 0 | | |  |
| PA-E3 | Burkholderiaceae | | T3.2 0-7cm | | 0.00 | | 0.00 | | | 0.02 | | 0 | | |  |
| PA-F3 | Burkholderiaceae | | T3.2 0-7cm | | 0.00 | | 0.00 | | | 0.02 | | 0 | | |  |
| PA-G3 | Burkholderiaceae | | T3.2 0-7cm | | 0.00 | | 0.25 | | | 0.68 | | 1 | | |  |
| PA-H3 | Burkholderiaceae | | T3.2 0-7cm | | 0.00 | | 0.04 | | | 0.25 | | 0 | | |  |
| PA-A4 | Burkholderiaceae | | T3.2 0-7cm | | 0.00 | | 0.08 | | | 0.07 | | 0 | | |  |
| PA-B4 | Burkholderiaceae | | T3.2 0-7cm | | 0.01 | | 0.00 | | | 0.46 | | 1 | | |  |
| PA-C4 | Burkholderiaceae | | T3.2 0-7cm | | 0.00 | | 0.02 | | | 0.76 | | 0 | | |  |
| PA-D4 | Burkholderiaceae | | T3.2 0-7cm | | 0.96 | | 0.05 | | | 0.79 | | 0 | | |  |
| PA-E4 | N.A | | T3.2 0-7cm | | 0.00 | | 0.01 | | | 0.01 | | 0 | | |  |
| PA-F4 | Burkholderiaceae | | T3.2 0-7cm | | 0.00 | | 0.19 | | | 1.05 | | 3 | | |  |
| PA-G4 | Burkholderiaceae | | T3.2 0-7cm | | 0.00 | | 0.17 | | | 0.65 | | 0 | | |  |
| PA-H4 | Burkholderiaceae | | T3.2 0-7cm | | -0.02 | | 0.00 | | | 0.01 | | 0 | | |  |
| PA-A5 | Burkholderiaceae | | T3.2 7-13.5cm | | 0.00 | | 0.02 | | | 0.01 | | 0 | | |  |
| PA-B5 | Methylobacteriaceae | | T3.2 7-13.5cm | | 0.00 | | 0.01 | | | 0.41 | | 0 | | |  |
| PA-C5 | Burkholderiaceae | | T3.2 7-13.5cm | | 0.72 | | 0.04 | | | 0.39 | | 0 | | |  |
| PA-D5 | Burkholderiaceae | | T3.2 7-13.5cm | | 1.04 | | 0.00 | | | 0.38 | | 0 | | |  |
| PA-E5 | Methylobacteriaceae | | T3.2 7-13.5cm | | 0.00 | | 0.01 | | | 0.00 | | 0 | | |  |
| PA-F5 | Burkholderiaceae | | T3.2 7-13.5cm | | -0.01 | | 0.01 | | | 0.42 | | 2 | | |  |
| PA-G5 | Burkholderiaceae | | T3.2 7-13.5cm | | 0.00 | | 0.00 | | | 0.44 | | 2 | | |  |
| PA-H5 | Burkholderiaceae | | T3.2 7-13.5cm | | 0.00 | | 0.05 | | | 0.45 | | 0 | | |  |
| PA-A6 | Burkholderiaceae | | T3.2 7-13.5cm | | 0.00 | | 0.00 | | | 0.34 | | 2 | | |  |
| PA-B6 | Burkholderiaceae | | T3.2 7-13.5cm | | 0.00 | | 0.01 | | | 0.28 | | 3 | | |  |
| PA-C6 | Burkholderiaceae | | T3.2 7-13.5cm | | 0.00 | | 0.04 | | | 0.41 | | 1 | | |  |
| PA-D6 | Burkholderiaceae | | T3.2 7-13.5cm | | 0.00 | | 0.04 | | | 0.62 | | 2 | | |  |
| PA-E6 | Burkholderiaceae | | T3.2 7-13.5cm | | 0.00 | | 0.00 | | | 0.44 | | 2 | | |  |
| PA-F6 | Burkholderiaceae | | T3.2 7-13.5cm | | 0.00 | | 0.05 | | | 0.10 | | 0 | | |  |
| PA-G6 | Burkholderiaceae | | T3.2 7-13.5cm | | 0.00 | | 0.04 | | | 0.42 | | 0 | | |  |
| PA-H6 | Burkholderiaceae | | T3.2 7-13.5cm | | 0.00 | | 0.00 | | | 0.43 | | 1 | | |  |
| PA-A7 | Burkholderiaceae | | T3.2 13.5-20cm | | 0.00 | | 0.15 | | | 0.57 | | 1 | | |  |
| PA-B7 | Burkholderiaceae | | T3.2 13.5-20cm | | 0.91 | | 0.09 | | | 0.50 | | 0 | | |  |
| PA-C7 | Burkholderiaceae | | T3.2 13.5-20cm | | 0.00 | | 0.01 | | | 0.81 | | 2 | | |  |
| PA-D7 | Burkholderiaceae | | T3.2 13.5-20cm | | 1.36 | | 0.01 | | | 0.98 | | 2 | | |  |
| PA-E7 | Burkholderiaceae | | T3.2 13.5-20cm | | 0.00 | | 0.02 | | | 0.69 | | 3 | | |  |
| PA-F7 | Burkholderiaceae | | T3.2 13.5-20cm | | 0.00 | | 0.06 | | | 0.57 | | 0 | | |  |
| PA-G7 | Burkholderiaceae | | T3.2 13.5-20cm | | 0.00 | | 0.05 | | | 0.72 | | 0 | | |  |
| PA-H7 | Burkholderiaceae | | T3.2 13.5-20cm | | 0.00 | | 0.06 | | | 1.24 | | 0 | | |  |
| PA-A8 | Burkholderiaceae | | T3.2 13.5-20cm | | 0.00 | | 0.09 | | | 1.36 | | 0 | | |  |
| PA-B8 | Burkholderiaceae | | T3.2 13.5-20cm | | 1.30 | | 0.02 | | | 0.82 | | 3 | | |  |
| PA-C8 | Burkholderiaceae | | T3.2 13.5-20cm | | 0.00 | | 0.01 | | | 0.90 | | 0 | | |  |
| PA-D8 | Burkholderiaceae | | T3.2 13.5-20cm | | 0.00 | | 0.02 | | | 1.23 | | 1 | | |  |
| PA-E8 | Burkholderiaceae | | T3.2 13.5-20cm | | 0.00 | | 0.18 | | | 1.16 | | 2 | | |  |
| PA-F8 | Burkholderiaceae | | T3.2 13.5-20cm | | 0.00 | | 0.01 | | | 0.82 | | 0 | | |  |
| PA-G8 | Burkholderiaceae | | T3.2 13.5-20cm | | 0.00 | | 0.01 | | | 0.93 | | 0 | | |  |
| PA-H8 | Burkholderiaceae | | T3.2 13.5-20cm | | 0.01 | | 0.01 | | | 0.74 | | 1 | | |  |
| PA-A9 | Burkholderiaceae | | T3.2 20-24.5 cm | | 0.00 | | 0.01 | | | 1.05 | | 0 | | |  |
| PA-B9 | Burkholderiaceae | | T3.2 20-24.5 cm | | 0.00 | | 0.04 | | | 0.99 | | 0 | | |  |
| PA-C9 | Burkholderiaceae | | T3.2 20-24.5 cm | | 0.00 | | 0.14 | | | 0.74 | | 0 | | |  |
| PA-D9 | Burkholderiaceae | | T3.2 20-24.5 cm | | 0.01 | | 0.03 | | | 1.13 | | 0 | | |  |
| PA-E9 | Burkholderiaceae | | T3.2 20-24.5 cm | | 0.00 | | 0.01 | | | 1.05 | | 0 | | |  |
| PA-F9 | Burkholderiaceae | | T3.2 20-24.5 cm | | 0.01 | | 0.22 | | | 0.62 | | 3 | | |  |
| PA-G9 | Burkholderiaceae | | T3.2 20-24.5 cm | | 0.00 | | 0.01 | | | 0.50 | | 1 | | |  |
| PA-H9 | Burkholderiaceae | | T3.2 20-24.5 cm | | 0.00 | | 0.01 | | | 0.82 | | 0 | | |  |
| PA-A10 | Burkholderiaceae | | T3.2 20-24.5 cm | | 0.00 | | 0.01 | | | 1.23 | | 0 | | |  |
| PA-B10 | Burkholderiaceae | | T3.2 20-24.5 cm | | 0.01 | | 0.01 | | | 0.85 | | 0 | | |  |
| PA-C10 | Burkholderiaceae | | T3.2 20-24.5 cm | | 0.00 | | 0.27 | | | 0.78 | | 3 | | |  |
| PA-D10 | N.A | | T3.2 20-24.5 cm | | 0.35 | | 0.00 | | | 0.67 | | 1 | | |  |
| PA-E10 | N.A | | T3.2 20-24.5 cm | | 0.00 | | 0.05 | | | 0.03 | | 0 | | |  |
| PA-F10 | N.A | | T3.2 20-24.5 cm | | -0.07 | | 0.06 | | | 0.26 | | 0 | | |  |
| PA-G10 | N.A | | T3.2 20-24.5 cm | | -0.07 | | 0.06 | | | 0.25 | | 0 | | |  |
| PA-H10 | Pseudomonadaceae | | T3.2 20-24.5 cm | | -0.06 | | 0.01 | | | 0.63 | | 0 | | |  |
| PA-A11 | Burkholderiaceae | | T3.2 24.5-END | | 0.00 | | 0.01 | | | 1.10 | | 0 | | |  |
| PA-B11 | Burkholderiaceae | | T3.2 24.5-END | | 0.00 | | 0.01 | | | 0.57 | | 0 | | |  |
| PA-C11 | Burkholderiaceae | | T3.2 24.5-END | | 0.00 | | 0.24 | | | 0.84 | | 3 | | |  |
| PA-D11 | N.A | | T3.2 24.5-END | | -0.01 | | 0.08 | | | 0.25 | | 0 | | |  |
| PA-E11 | Burkholderiaceae | | T3.2 24.5-END | | 0.00 | | 0.01 | | | 0.56 | | 0 | | |  |
| PA-F11 | Burkholderiaceae | | T3.2 24.5-END | | 0.00 | | 0.22 | | | 1.15 | | 1 | | |  |
| PA-G11 | Burkholderiaceae | | T3.2 24.5-END | | 0.00 | | 0.04 | | | 0.71 | | 0 | | |  |
| PA-H11 | Pseudomonadaceae | | T3.2 24.5-END | | 0.00 | | 0.01 | | | 0.34 | | 0 | | |  |
| PA-A12 | Burkholderiaceae | | T3.2 24.5-END | | 0.00 | | 0.02 | | | 1.23 | | 0 | | |  |
| PA-B12 | Burkholderiaceae | | T3.2 24.5-END | | 0.00 | | 0.34 | | | 1.42 | | 2 | | |  |
| PA-C12 | Burkholderiaceae | | T3.2 24.5-END | | 0.00 | | 0.01 | | | 1.21 | | 0 | | |  |
| PA-D12 | Burkholderiaceae | | T3.2 24.5-END | | 0.00 | | 0.01 | | | 0.84 | | 0 | | |  |
| PA-E12 | N.A | | T3.2 24.5-END | | 0.00 | | 0.06 | | | 0.05 | | 0 | | |  |
| PA-F12 | Burkholderiaceae | | T3.2 24.5-END | | 0.00 | | 0.01 | | | 0.77 | | 0 | | |  |
| PA-G12 | Burkholderiaceae | | T3.2 24.5-END | | 0.00 | | 0.10 | | | 0.85 | | 0 | | |  |
| PA-H12 | Burkholderiaceae | | T3.2 24.5-END | | 0.00 | | 0.22 | | | 1.24 | | 1 | | |  |
| AL-A1 | Oxalobacteraceae | | T1.3 | | 0.17 | | 0.04 | | | 1.6 | | 1 | | |  |
| AL-B1 | N.A | | T1.3 | | 0.01 | | 0.00 | | | -0.08 | | 0 | | |  |
| AL-C1 | Burkholderiaceae | | T1.3 | | 0.01 | | 0.00 | | | 1.49 | | 0 | | |  |
| AL-D1 | Burkholderiaceae | | T1.3 | | 0.01 | | 0.00 | | | 1.78 | | 0 | | |  |
| AL-E1 | Burkholderiaceae | | T1.3 | | 0.01 | | 0.00 | | | 1.76 | | 3 | | |  |
| AL-F1 | Burkholderiaceae | | T1.3 | | 0.01 | | 0.02 | | | 2.34 | | 3 | | |  |
| AL-G1 | Burkholderiaceae | | T1.3 | | 0.01 | | 0.00 | | | 2.28 | | 0 | | |  |
| AL-H1 | Burkholderiaceae | | T1.3 | | 0.01 | | 0.04 | | | 2.49 | | 3 | | |  |
| AL-A2 | Oxalobacteraceae | | T2.3 | | 0.21 | | 0.00 | | | 0.51 | | 0 | | |  |
| AL-B2 | Burkholderiaceae | | T2.3 | | 0.01 | | 0.00 | | | 0.2 | | 0 | | |  |
| AL-C2 | Burkholderiaceae | | T2.3 | | 0.01 | | 0.00 | | | 1.18 | | 0 | | |  |
| AL-D2 | Burkholderiaceae | | T2.3 | | 0.01 | | 0.01 | | | 2.05 | | 0 | | |  |
| AL-E2 | Burkholderiaceae | | T2.3 | | 0.01 | | 0.00 | | | 0.94 | | 0 | | |  |
| AL-F2 | micrococcaceae | | T2.3 | | 0.01 | | 0.00 | | | 0.64 | | 0 | | |  |
| AL-G2 | Burkholderiaceae | | T2.3 | | 0.01 | | 0.01 | | | 2.15 | | 0 | | |  |
| AL-H2 | Burkholderiaceae | | T2.3 | | 0.01 | | 0.00 | | | 1.88 | | 1 | | |  |
| AL-A3 | Burkholderiaceae | | T3.2 0-7cm | | 0.15 | | 0.00 | | | 1.21 | | 1 | | |  |
| AL-B3 | Burkholderiaceae | | T3.2 0-7cm | | -0.01 | | 0.00 | | | 0.73 | | 2 | | |  |
| AL-C3 | Burkholderiaceae | | T3.2 0-7cm | | 0.01 | | 0.00 | | | 1.13 | | 0 | | |  |
| AL-D3 | Burkholderiaceae | | T3.2 0-7cm | | 0.01 | | 0.00 | | | 1.34 | | 1 | | |  |
| AL-E3 | Burkholderiaceae | | T3.2 0-7cm | | 0.01 | | 0.00 | | | 1.68 | | 0 | | |  |
| AL-F3 | Burkholderiaceae | | T3.2 0-7cm | | 0.00 | | 0.00 | | | 1.07 | | 3 | | |  |
| AL-G3 | Burkholderiaceae | | T3.2 0-7cm | | 0.01 | | 0.00 | | | 1.49 | | 1 | | |  |
| AL-H3 | Burkholderiaceae | | T3.2 0-7cm | | -0.01 | | 0.00 | | | 2.03 | | 1 | | |  |
| AL-A4 | Streptomycetaceae | | T3.2 0-7cm | | 0.00 | | 0.00 | | | 0.7 | | 0 | | |  |
| AL-B4 | Burkholderiaceae | | T3.2 0-7cm | | 0.00 | | 0.00 | | | 0.15 | | 2 | | |  |
| AL-C4 | Burkholderiaceae | | T3.2 0-7cm | | 0.01 | | 0.00 | | | 0.59 | | 0 | | |  |
| AL-D4 | Burkholderiaceae | | T3.2 0-7cm | | 0.01 | | 0.03 | | | 1.88 | | 0 | | |  |
| AL-E4 | Burkholderiaceae | | T3.2 0-7cm | | 0.01 | | 0.00 | | | 0.98 | | 0 | | |  |
| AL-F4 | Burkholderiaceae | | T3.2 0-7cm | | 0.00 | | 0.00 | | | 1.86 | | 0 | | |  |
| AL-G4 | Streptomycetaceae | | T3.2 0-7cm | | 0.00 | | 0.00 | | | 1.78 | | 0 | | |  |
| AL-H4 | Streptomycetaceae | | T3.2 0-7cm | | 0.00 | | 0.00 | | | 1.5 | | 0 | | |  |
| AL-A5 | Pseudomonadaceae | | T3.2 7-13.5cm | | 0.01 | | 0.00 | | | 0.48 | | 3 | | |  |
| AL-B5 | Burkholderiaceae | | T3.2 7-13.5cm | | 0.01 | | 0.00 | | | 0.71 | | 1 | | |  |
| AL-C5 | Burkholderiaceae | | T3.2 7-13.5cm | | 0.01 | | 0.00 | | | 0.67 | | 0 | | |  |
| AL-D5 | Burkholderiaceae | | T3.2 7-13.5cm | | 0.01 | | 0.00 | | | 2.13 | | 0 | | |  |
| AL-E5 | Burkholderiaceae | | T3.2 7-13.5cm | | 0.03 | | 0.00 | | | 0.92 | | 0 | | |  |
| AL-F5 | Pseudomonadaceae | | T3.2 7-13.5cm | | 0.01 | | 0.00 | | | 1.14 | | 3 | | |  |
| AL-G5 | Burkholderiaceae | | T3.2 7-13.5cm | | 0.01 | | 0.01 | | | 2.63 | | 0 | | |  |
| AL-H5 | Burkholderiaceae | | T3.2 7-13.5cm | | 0.01 | | 0.01 | | | 2.32 | | 3 | | |  |
| AL-A6 | Burkholderiaceae | | T3.2 7-13.5cm | | 0.01 | | 0.01 | | | 1.3 | | 3 | | |  |
| AL-B6 | Pseudomonadaceae | | T3.2 7-13.5cm | | 0.01 | | 0.00 | | | 0.45 | | 3 | | |  |
| AL-C6 | Burkholderiaceae | | T3.2 7-13.5cm | | 0.01 | | 0.00 | | | 2.16 | | 0 | | |  |
| AL-D6 | Burkholderiaceae | | T3.2 7-13.5cm | | 0.26 | | 0.09 | | | 2.36 | | 0 | | |  |
| AL-E6 | Burkholderiaceae | | T3.2 7-13.5cm | | 0.01 | | 0.02 | | | 1.68 | | 2 | | |  |
| AL-F6 | Burkholderiaceae | | T3.2 7-13.5cm | | 0.01 | | 0.00 | | | 1.6 | | 0 | | |  |
| AL-G6 | Burkholderiaceae | | T3.2 7-13.5cm | | 0.02 | | 0.00 | | | 2.8 | | 0 | | |  |
| AL-H6 | Burkholderiaceae | | T3.2 7-13.5cm | | 0.01 | | 0.01 | | | 2.44 | | 3 | | |  |
| AL-A7 | Burkholderiaceae | | T3.2 13.5-20cm | | 0.01 | | 0.00 | | | 2.09 | | 0 | | |  |
| AL-B7 | Burkholderiaceae | | T3.2 13.5-20cm | | 0.00 | | 0.00 | | | 1.35 | | 0 | | |  |
| AL-C7 | Burkholderiaceae | | T3.2 13.5-20cm | | 0.01 | | 0.00 | | | 0.98 | | 1 | | |  |
| AL-D7 | Burkholderiaceae | | T3.2 13.5-20cm | | 0.00 | | 0.00 | | | 1.01 | | 1 | | |  |
| AL-E7 | Burkholderiaceae | | T3.2 13.5-20cm | | 0.00 | | 0.00 | | | 1.31 | | 0 | | |  |
| AL-F7 | Burkholderiaceae | | T3.2 13.5-20cm | | 0.01 | | 0.00 | | | 1.59 | | 0 | | |  |
| AL-G7 | Burkholderiaceae | | T3.2 13.5-20cm | | 0.01 | | 0.00 | | | 1.71 | | 0 | | |  |
| AL-H7 | Burkholderiaceae | | T3.2 13.5-20cm | | 0.00 | | 0.08 | | | 1.64 | | 1 | | |  |
| AL-A8 | Burkholderiaceae | | T3.2 13.5-20cm | | 0.02 | | 0.00 | | | 1.47 | | 0 | | |  |
| AL-B8 | Burkholderiaceae | | T3.2 13.5-20cm | | 0.01 | | 0.01 | | | 2.27 | | 1 | | |  |
| AL-C8 | Burkholderiaceae | | T3.2 13.5-20cm | | 0.00 | | 0.00 | | | 1.73 | | 1 | | |  |
| AL-D8 | Burkholderiaceae | | T3.2 13.5-20cm | | 0.00 | | 0.00 | | | 1.29 | | 2 | | |  |
| AL-E8 | Burkholderiaceae | | T3.2 13.5-20cm | | 0.00 | | 0.00 | | | 1.37 | | 0 | | |  |
| AL-F8 | Burkholderiaceae | | T3.2 13.5-20cm | | 0.01 | | 0.01 | | | 1.25 | | 0 | | |  |
| AL-G8 | Burkholderiaceae | | T3.2 13.5-20cm | | 0.00 | | 0.00 | | | 1.08 | | 1 | | |  |
| AL-H8 | Burkholderiaceae | | T3.2 13.5-20cm | | 0.01 | | 0.00 | | | 0.37 | | 1 | | |  |
| AL-A9 | Burkholderiaceae | | T3.2 20-24.5 cm | | 0.02 | | 0.00 | | | 0.82 | | 0 | | |  |
| AL-B9 | Burkholderiaceae | | T3.2 20-24.5 cm | | 0.01 | | 0.00 | | | 0.56 | | 0 | | |  |
| AL-C9 | Burkholderiaceae | | T3.2 20-24.5 cm | | -0.02 | | 0.00 | | | 0.57 | | 0 | | |  |
| AL-D9 | Burkholderiaceae | | T3.2 20-24.5 cm | | 0.01 | | 0.00 | | | 1.92 | | 0 | | |  |
| AL-E9 | Burkholderiaceae | | T3.2 20-24.5 cm | | -0.04 | | 0.00 | | | 1.33 | | 1 | | |  |
| AL-F9 | Burkholderiaceae | | T3.2 20-24.5 cm | | 0.33 | | 0.01 | | | 1.17 | | 3 | | |  |
| AL-G9 | Burkholderiaceae | | T3.2 20-24.5 cm | | 0.00 | | 0.00 | | | 1.5 | | 0 | | |  |
| AL-H9 | Burkholderiaceae | | T3.2 20-24.5 cm | | 0.06 | | 0.02 | | | 1.18 | | 2 | | |  |
| AL-A10 | Burkholderiaceae | | T3.2 20-24.5 cm | | 0.00 | | 0.00 | | | 0.8 | | 1 | | |  |
| AL-B10 | Burkholderiaceae | | T3.2 20-24.5 cm | | 0.00 | | 0.00 | | | 0.81 | | 0 | | |  |
| AL-C10 | Burkholderiaceae | | T3.2 20-24.5 cm | | 0.01 | | 0.00 | | | 1.19 | | 0 | | |  |
| AL-D10 | Burkholderiaceae | | T3.2 20-24.5 cm | | 0.01 | | 0.00 | | | 1.1 | | 0 | | |  |
| AL-E10 | Burkholderiaceae | | T3.2 20-24.5 cm | | 0.00 | | 0.00 | | | 0.98 | | 0 | | |  |
| AL-F10 | Burkholderiaceae | | T3.2 20-24.5 cm | | 0.00 | | -0.02 | | | 1.16 | | 0 | | |  |
| AL-G10 | Burkholderiaceae | | T3.2 20-24.5 cm | | 0.00 | | -0.01 | | | 1.31 | | 0 | | |  |
| AL-H10 | Burkholderiaceae | | T3.2 20-24.5 cm | | 0.01 | | -0.02 | | | 0.71 | | 1 | | |  |
| AL-A11 | Burkholderiaceae | | T3.2 24.5-END | | 0.01 | | 0.00 | | | 1.66 | | 0 | | |  |
| AL-B11 | Burkholderiaceae | | T3.2 24.5-END | | 0.01 | | 0.01 | | | 1.89 | | 0 | | |  |
| AL-C11 | Burkholderiaceae | | T3.2 24.5-END | | 0.01 | | 0.00 | | | 1.33 | | 0 | | |  |
| AL-D11 | Burkholderiaceae | | T3.2 24.5-END | | -0.01 | | 0.00 | | | 1.42 | | 0 | | |  |
| AL-E11 | Burkholderiaceae | | T3.2 24.5-END | | 0.53 | | 0.07 | | | 1.39 | | 0 | | |  |
| AL-F11 | Burkholderiaceae | | T3.2 24.5-END | | 0.01 | | 0.01 | | | 1.04 | | 3 | | |  |
| AL-G11 | Streptomycetaceae | | T3.2 24.5-END | | 0.01 | | 0.00 | | | 0.69 | | 0 | | |  |
| AL-H11 | Burkholderiaceae | | T3.2 24.5-END | | 0.01 | | 0.02 | | | 0.68 | | 0 | | |  |
| AL-A12 | Burkholderiaceae | | T3.2 24.5-END | | 0.01 | | 0.00 | | | 1.32 | | 0 | | |  |
| AL-B12 | Streptomycetaceae | | T3.2 24.5-END | | 0.44 | | 0.01 | | | 0.21 | | 0 | | |  |
| AL-C12 | Burkholderiaceae | | T3.2 24.5-END | | 0.01 | | 0.01 | | | 0.78 | | 0 | | |  |
| AL-D12 | Burkholderiaceae | | T3.2 24.5-END | | 0.01 | | 0.01 | | | 2.42 | | 0 | | |  |
| AL-E12 | Burkholderiaceae | | T3.2 24.5-END | | 0.01 | | 0.01 | | | 1.28 | | 0 | | |  |
| AL-F12 | Burkholderiaceae | | T3.2 24.5-END | | 0.01 | | 0.00 | | | 1.66 | | 0 | | |  |
| AL-G12 | Burkholderiaceae | | T3.2 24.5-END | | 0.01 | | 0.00 | | | 1.06 | | 0 | | |  |
| AL-H12 | Burkholderiaceae | | T3.2 24.5-END | | 0.01 | | 0.00 | | | 1.04 | | 1 | | |  |
| FE-A1 | Burkholderiaceae | | T1.3 | | 0.01 | | 0.01 | | | 1.82 | | 2 | | |  |
| FE-B1 | Burkholderiaceae | | T1.3 | | 0.01 | | 0.01 | | | 0.01 | | 0 | | |  |
| FE-C1 | N.A | | T1.3 | | 0.04 | | 0.04 | | | 0.17 | | 0 | | |  |
| FE-D1 | N.A | | T1.3 | | 0.00 | | 0.00 | | | 0.65 | | 1 | | |  |
| FE-E1 | Burkholderiaceae | | T1.3 | | 0.00 | | 0.00 | | | 0.79 | | 0 | | |  |
| FE-F1 | Burkholderiaceae | | T1.3 | | 0.00 | | 0.00 | | | 0.76 | | 0 | | |  |
| FE-G1 | Burkholderiaceae | | T1.3 | | 0.00 | | 0.00 | | | 1.24 | | 1 | | |  |
| FE-H1 | Burkholderiaceae | | T1.3 | | 0.08 | | 0.08 | | | 0.41 | | 2 | | |  |
| FE-A2 | Burkholderiaceae | | T2.3 | | 0.01 | | 0.01 | | | 0.68 | | 0 | | |  |
| FE-B2 | phyllobacteriaceae | | T2.3 | | 0.01 | | 0.01 | | | 0.35 | | 0 | | |  |
| FE-C2 | Burkholderiaceae | | T2.3 | | 0.01 | | 0.02 | | | 0.76 | | 0 | | |  |
| FE-D2 | Burkholderiaceae | | T2.3 | | 0.01 | | 0.01 | | | 0.69 | | 1 | | |  |
| FE-E2 | Burkholderiaceae | | T2.3 | | 0.01 | | 0.02 | | | 1.02 | | 0 | | |  |
| FE-F2 | Oxalobacteraceae | | T2.3 | | 0.04 | | 0.09 | | | 1.26 | | 1 | | |  |
| FE-G2 | Burkholderiaceae | | T2.3 | | 0.00 | | 0.01 | | | 0.71 | | 1 | | |  |
| FE-H2 | Burkholderiaceae | | T2.3 | | 0.01 | | 0.01 | | | 1.14 | | 1 | | |  |
| FE-A3 | Burkholderiaceae | | T3.2 0-7cm | | 0.01 | | 0.01 | | | 1.04 | | 2 | | |  |
| FE-B3 | Burkholderiaceae | | T3.2 0-7cm | | 0.04 | | 0.06 | | | 0.17 | | 0 | | |  |
| FE-C3 | Burkholderiaceae | | T3.2 0-7cm | | 0.01 | | 0.01 | | | 0.96 | | 2 | | |  |
| FE-D3 | Burkholderiaceae | | T3.2 0-7cm | | 0.01 | | 0.01 | | | 0.89 | | 0 | | |  |
| FE-E3 | Burkholderiaceae | | T3.2 0-7cm | | 0.40 | | 0.01 | | | 1.48 | | 2 | | |  |
| FE-F3 | Burkholderiaceae | | T3.2 0-7cm | | -0.01 | | 0.02 | | | 1.29 | | 3 | | |  |
| FE-G3 | Burkholderiaceae | | T3.2 0-7cm | | 0.01 | | 0.01 | | | 0.73 | | 3 | | |  |
| FE-H3 | Burkholderiaceae | | T3.2 0-7cm | | 0.01 | | 0.01 | | | 1.02 | | 0 | | |  |
| FE-A4 | Burkholderiaceae | | T3.2 0-7cm | | -0.01 | | 0.01 | | | 0.83 | | 0 | | |  |
| FE-B4 | Burkholderiaceae | | T3.2 0-7cm | | -0.01 | | 0.01 | | | 0.87 | | 2 | | |  |
| FE-C4 | Burkholderiaceae | | T3.2 0-7cm | | 0.00 | | 0.01 | | | 0.80 | | 2 | | |  |
| FE-D4 | Burkholderiaceae | | T3.2 0-7cm | | 0.00 | | 0.01 | | | 0.66 | | 2 | | |  |
| FE-E4 | Burkholderiaceae | | T3.2 0-7cm | | 0.00 | | 0.01 | | | 1.03 | | 0 | | |  |
| FE-F4 | Burkholderiaceae | | T3.2 0-7cm | | 0.01 | | 0.01 | | | 0.49 | | 0 | | |  |
| FE-G4 | Burkholderiaceae | | T3.2 0-7cm | | 0.00 | | 0.02 | | | 1.36 | | 3 | | |  |
| FE-H4 | Burkholderiaceae | | T3.2 0-7cm | | 0.02 | | 0.01 | | | 1.25 | | 1 | | |  |
| FE-A5 | Burkholderiaceae | | T3.2 7-13.5cm | | 0.01 | | 0.02 | | | 1.40 | | 0 | | |  |
| FE-B5 | Burkholderiaceae | | T3.2 7-13.5cm | | 0.54 | | 0.01 | | | 0.21 | | 0 | | |  |
| FE-C5 | Burkholderiaceae | | T3.2 7-13.5cm | | 0.00 | | 0.01 | | | 0.74 | | 3 | | |  |
| FE-D5 | Burkholderiaceae | | T3.2 7-13.5cm | | 0.00 | | 0.01 | | | 1.22 | | 0 | | |  |
| FE-E5 | Burkholderiaceae | | T3.2 7-13.5cm | | 0.01 | | 0.01 | | | 0.65 | | 0 | | |  |
| FE-F5 | Burkholderiaceae | | T3.2 7-13.5cm | | 0.01 | | 0.02 | | | 0.94 | | 2 | | |  |
| FE-G5 | Burkholderiaceae | | T3.2 7-13.5cm | | 0.01 | | 0.02 | | | 0.99 | | 2 | | |  |
| FE-H5 | Burkholderiaceae | | T3.2 7-13.5cm | | 0.01 | | 0.01 | | | 0.92 | | 0 | | |  |
| FE-A6 | Burkholderiaceae | | T3.2 7-13.5cm | | 0.00 | | 0.01 | | | 0.07 | | 0 | | |  |
| FE-B6 | Burkholderiaceae | | T3.2 7-13.5cm | | 0.00 | | 0.02 | | | 1.41 | | 0 | | |  |
| FE-C6 | Burkholderiaceae | | T3.2 7-13.5cm | | 0.00 | | 0.02 | | | 1.40 | | 0 | | |  |
| FE-D6 | Burkholderiaceae | | T3.2 7-13.5cm | | 0.01 | | 0.02 | | | 1.54 | | 0 | | |  |
| FE-E6 | Burkholderiaceae | | T3.2 7-13.5cm | | 0.00 | | 0.01 | | | 1.28 | | 0 | | |  |
| FE-F6 | Burkholderiaceae | | T3.2 7-13.5cm | | 0.00 | | 0.01 | | | 0.39 | | 0 | | |  |
| FE-G6 | Burkholderiaceae | | T3.2 7-13.5cm | | 0.01 | | 0.01 | | | 1.06 | | 3 | | |  |
| FE-H6 | Burkholderiaceae | | T3.2 7-13.5cm | | 0.01 | | 0.01 | | | 1.36 | | 1 | | |  |
| FE-A7 | Burkholderiaceae | | T3.2 13.5-20cm | | -0.01 | | 0 | | | 1.71 | | 0 | | |  |
| FE-B7 | Burkholderiaceae | | T3.2 13.5-20cm | | -0.01 | | 0.01 | | | 0.53 | | 2 | | |  |
| FE-C7 | Burkholderiaceae | | T3.2 13.5-20cm | | 0.05 | | 0.07 | | | 0.36 | | 0 | | |  |
| FE-D7 | Burkholderiaceae | | T3.2 13.5-20cm | | -0.01 | | 0.01 | | | 0.62 | | 0 | | |  |
| FE-E7 | Burkholderiaceae | | T3.2 13.5-20cm | | -0.01 | | 0.01 | | | 1.61 | | 0 | | |  |
| FE-F7 | Burkholderiaceae | | T3.2 13.5-20cm | | 0.00 | | 0.02 | | | 0.34 | | 0 | | |  |
| FE-G7 | Pseudomonadaceae | | T3.2 13.5-20cm | | -0.01 | | 0.01 | | | 0.17 | | 2 | | |  |
| FE-H7 | Pseudomonadaceae | | T3.2 13.5-20cm | | 0.03 | | 0.07 | | | 0.15 | | 0 | | |  |
| FE-A8 | Burkholderiaceae | | T3.2 13.5-20cm | | -0.01 | | 0.05 | | | 1.35 | | 3 | | |  |
| FE-B8 | Burkholderiaceae | | T3.2 13.5-20cm | | -0.01 | | 0.06 | | | 1.22 | | 0 | | |  |
| FE-C8 | Burkholderiaceae | | T3.2 13.5-20cm | | -0.02 | | 0.02 | | | 1.38 | | 0 | | |  |
| FE-D8 | Burkholderiaceae | | T3.2 13.5-20cm | | -0.02 | | 0.01 | | | 1.01 | | 0 | | |  |
| FE-E8 | Burkholderiaceae | | T3.2 13.5-20cm | | -0.01 | | 0.04 | | | 1.23 | | 0 | | |  |
| FE-F8 | Burkholderiaceae | | T3.2 13.5-20cm | | -0.01 | | 0.05 | | | 0.75 | | 0 | | |  |
| FE-G8 | Burkholderiaceae | | T3.2 13.5-20cm | | -0.02 | | 0.02 | | | 0.61 | | 2 | | |  |
| FE-H8 | Burkholderiaceae | | T3.2 13.5-20cm | | -0.01 | | 0.01 | | | 0.29 | | 2 | | |  |
| FE-A9 | Burkholderiaceae | | T3.2 20-24.5 cm | | -0.01 | | 0.04 | | | 1.91 | | 2 | | |  |
| FE-B9 | Burkholderiaceae | | T3.2 20-24.5 cm | | -0.01 | | 0.01 | | | 1.26 | | 0 | | |  |
| FE-C9 | Burkholderiaceae | | T3.2 20-24.5 cm | | 0.00 | | 0.01 | | | 1.45 | | 0 | | |  |
| FE-D9 | Burkholderiaceae | | T3.2 20-24.5 cm | | -0.01 | | 0.09 | | | 1.69 | | 0 | | |  |
| FE-E9 | Burkholderiaceae | | T3.2 20-24.5 cm | | -0.01 | | 0.01 | | | 1.44 | | 0 | | |  |
| FE-F9 | Burkholderiaceae | | T3.2 20-24.5 cm | | 0.00 | | 0.06 | | | 1.04 | | 0 | | |  |
| FE-G9 | Burkholderiaceae | | T3.2 20-24.5 cm | | -0.01 | | 0.03 | | | 0.73 | | 0 | | |  |
| FE-H9 | Burkholderiaceae | | T3.2 20-24.5 cm | | -0.01 | | 0.01 | | | 0.53 | | 1 | | |  |
| FE-A10 | Burkholderiaceae | | T3.2 20-24.5 cm | | -0.01 | | 0.02 | | | 1.27 | | 0 | | |  |
| FE-B10 | Burkholderiaceae | | T3.2 20-24.5 cm | | -0.02 | | 0.02 | | | 1.20 | | 2 | | |  |
| FE-C10 | Burkholderiaceae | | T3.2 20-24.5 cm | | -0.01 | | 0.02 | | | 1.53 | | 1 | | |  |
| FE-D10 | Burkholderiaceae | | T3.2 20-24.5 cm | | -0.01 | | 0.07 | | | 1.16 | | 2 | | |  |
| FE-E10 | N.A | | T3.2 20-24.5 cm | | -0.01 | | 0.01 | | | 0.11 | | 1 | | |  |
| FE-F10 | Burkholderiaceae | | T3.2 20-24.5 cm | | -0.01 | | 0.01 | | | 0.08 | | 1 | | |  |
| FE-G10 | Burkholderiaceae | | T3.2 20-24.5 cm | | -0.01 | | 0 | | | 0.72 | | 0 | | |  |
| FE-H10 | Burkholderiaceae | | T3.2 20-24.5 cm | | -0.01 | | 0.01 | | | 0.03 | | 2 | | |  |
| FE-A11 | Burkholderiaceae | | T3.2 24.5-END | | -0.01 | | 0.01 | | | 0.92 | | 0 | | |  |
| FE-B11 | Burkholderiaceae | | T3.2 24.5-END | | -0.02 | | 0.07 | | | 1.64 | | 2 | | |  |
| FE-C11 | Burkholderiaceae | | T3.2 24.5-END | | -0.01 | | 0.01 | | | 0.50 | | 2 | | |  |
| FE-D11 | Burkholderiaceae | | T3.2 24.5-END | | 0.00 | | 0.01 | | | 1.01 | | 0 | | |  |
| FE-E11 | Burkholderiaceae | | T3.2 24.5-END | | -0.03 | | 0.01 | | | 1.00 | | 0 | | |  |
| FE-F11 | Burkholderiaceae | | T3.2 24.5-END | | -0.01 | | 0.01 | | | 0.92 | | 0 | | |  |
| FE-G11 | Burkholderiaceae | | T3.2 24.5-END | | -0.01 | | 0.02 | | | 0.18 | | 2 | | |  |
| FE-H11 | Burkholderiaceae | | T3.2 24.5-END | | -0.02 | | 0.16 | | | 0.83 | | 2 | | |  |
| FE-A12 | Burkholderiaceae | | T3.2 24.5-END | | -0.01 | | 0.01 | | | 1.47 | | 0 | | |  |
| FE-B12 | Burkholderiaceae | | T3.2 24.5-END | | -0.01 | | 0.12 | | | 1.57 | | 1 | | |  |
| FE-C12 | Burkholderiaceae | | T3.2 24.5-END | | -0.03 | | 0.01 | | | 1.63 | | 0 | | |  |
| FE-D12 | Burkholderiaceae | | T3.2 24.5-END | | -0.01 | | 0.01 | | | 1.49 | | 0 | | |  |
| FE-E12 | Burkholderiaceae | | T3.2 24.5-END | | -0.01 | | 0.04 | | | 1.22 | | 0 | | |  |
| FE-F12 | Burkholderiaceae | | T3.2 24.5-END | | -0.01 | | 0.02 | | | 1.20 | | 0 | | |  |
| FE-G12 | Burkholderiaceae | | T3.2 24.5-END | | 0.00 | | 0.02 | | | 1.01 | | 0 | | |  |
| FE-H12 | Burkholderiaceae | | T3.2 24.5-END | | -0.01 | | 0.01 | | | 0.50 | | 0 | | |  |

**Supplementary Table S4.** The number of sequences loss during bioinformatics processing.

| Sample_name | Sample_ID | Demultiplexing | UParse/USearch | Rarefy |
| --- | --- | --- | --- | --- |
| T1.1 0 - 18cm | T1 | 352585 | 117198 | 12522 |
| T1.2 0 - 17.5cm | T2 | 569065 | 181240 | 12522 |
| T1.3 0 - 12cm | T3 | 467793 | 142598 | 12522 |
| T1.4 0 - 18cm | T4 | 757818 | 251523 | 12522 |
| T2.1 0 - 5cm | T5 | 730584 | 207447 | 12522 |
| T2.2 0 - 5cm | T6 | 1088132 | 398264 | 12522 |
| T2.3 0-5cm | T7 | 693162 | 226895 | 12522 |
| T2.4 0 - 6cm | T8 | 442193 | 133539 | 12522 |
| T3.1 0-6.5cm | T9 | 362025 | 118638 | 12522 |
| T3.1 6.5 - 15cm | T10 | 544805 | 174937 | 12522 |
| T3.1 15 - 21cm | T11 | 450677 | 136455 | 12522 |
| T3.1 21 - 25cm (end) | T12 | 599071 | 206420 | 12522 |
| T3.2 0 - 7cm | T13 | 485020 | 160652 | 12522 |
| T3.2 7-13.5cm | T14 | 482168 | 179387 | 12522 |
| T3.2 13.5 - 20cm | T15 | 576518 | 158140 | 12522 |
| T3.2 20 - 24.5cm | T16 | 444403 | 149915 | 12522 |
| T3.2 24.5cm - 30 | T17 | 818305 | 314102 | 12522 |
| T3.3 0 - 7cm | T18 | 692664 | 270877 | 12522 |
| T3.3 7 - 13.5cm | T19 | 441453 | 136921 | 12522 |
| T3.3 13.5 - 19cm | T20 | 901685 | 307404 | 12522 |
| T3.3 19 - 23cm | T21 | 347719 | 122007 | 12522 |
| T3.3 23 - 30cm | T22 | 752900 | 265950 | 12522 |
| T3.4 0 - 7cm | T23 | 507481 | 169201 | 12522 |
| T3.4 7 - 13.5cm | T24 | 548739 | 185466 | 12522 |
| T3.4 13.5 - 18.5cm | T25 | 455396 | 147764 | 12522 |
| T3.4 18.5 - 22.5cm | T26 | 703837 | 265875 | 12522 |
| T3.4 22.5 - 30cm | T27 | 35181 | 13914 | 12522 |
|  |  |  |  |  |
| Total number sequences written |  | 15251379 | 5142729 | 338094 |
