# Supplementary material for: Microbial Phosphorus Mobilization Strategies Across a Natural Nutrient Limitation Gradient and Evidence for Linkage With Iron Solubilization Traits
Source: Front Microbiol. 2021 Jun 23;12:572212. doi: 10.3389/fmicb.2021.572212 (PMC8261140; doi:10.3389/fmicb.2021.572212)
Supplement: Supplementary file 2 [file Table_1.DOCX]

*Supplemental methods for Wang et al “****Microbial phosphorus mobilization strategies across a natural nutrient limitation gradient and evidence for linkage with iron solubilization traits”***

*Quantitative PCR analysis*

The V3–V4 region of bacterial 16S rRNA gene was amplified using the primers 338F (5′- ACT CCT ACG GGA GGC AGC A-3′) and 806R (5′- GGA CTA CHV GGG TWT CTA AT-3′) (Mori et al. 2014). Bacterial amplification was completed with the following thermocycler settings, 95 °C for 3 min, 35 cycles of 95 °C for 30 s, 52 °C for 40 s, and 72°C for 90 s with a extension of 10 min at 72 °C. Then Melting curve 60°C to 95°C +2°C/min, fluorescence measured for 15 sec. Final hold at 25°C. Absolute quantifications for DNA samples were achieved by constructing standard curves with serial dilutions of linearized plasmids harboring PCR-amplified inserts of the targeted 16s rRNA genes. The pGEM-T Vector System II (Promega, Madison, WI, USA) was used for cloning and transformation into *Escherichia coli* JM109. The standard curves for qPCR, generated with tenfold dilutions (10^-1^-10^-7^) of plasmids harboring the target sequences, had a correlation coefficient r^2^ > 0.99 in the assays.
